# Therapeutic Properties and Biological Benefits of Marine-Derived Anticancer Peptides

**DOI:** 10.3390/ijms19030919

**Published:** 2018-03-20

**Authors:** Hee Kyoung Kang, Moon-Chang Choi, Chang Ho Seo, Yoonkyung Park

**Affiliations:** 1 Department of Biomedical Science, Chosun University, Gwangju 501-759, Korea; hkkang129@gmail.com (H.K.K.); choist777@gmail.com (M.-C.C.); 2 Department of Bioinformatics, Kongju National University, Kongju 314-701, Korea; chseo@kongju.ac.kr; 3 Research Center for Proteineous Materials, Chosun University, Gwangju 501-759, Korea

**Keywords:** peptide, anticancer, marine organism, antiproliferative, therapeutic agents

## Abstract

Various organisms exist in the oceanic environment. These marine organisms provide an abundant source of potential medicines. Many marine peptides possess anticancer properties, some of which have been evaluated for treatment of human cancer in clinical trials. Marine anticancer peptides kill cancer cells through different mechanisms, such as apoptosis, disruption of the tubulin-microtubule balance, and inhibition of angiogenesis. Traditional chemotherapeutic agents have side effects and depress immune responses. Thus, the research and development of novel anticancer peptides with low toxicity to normal human cells and mechanisms of action capable of avoiding multi-drug resistance may provide a new method for anticancer treatment. This review provides useful information on the potential of marine anticancer peptides for human therapy.

## 1. Introduction

The oceans are a diverse environment comprising about 75% of living organisms [1]. The oceanic environment is an abundant source of nutraceuticals and potential candidates with therapeutic functions [2]. 

Cancer is among the major causes of death with high morbidity and mortality [3]. Cell division that occurs within the cell tissue is a normal process. Under normal conditions, apoptosis constantly maintains the equilibrium between proliferating cells and programmed cell death. Additionally, DNA mutations cause cancer by interruption of the regulating programs. In carcinogenesis, normal cells are transformed into cancer cells [4,5]. In recent decades, marine compounds were studied for the treatment of cancer. Various types of marine products, such as alkaloids, polyketides, terpenes, peptides, and carbohydrates, have the potential to prevent and cure cancer [6]. Therefore, these products may be important in the development of anticancer drugs [7].

In recent years, novel peptides with antibacterial, antidiabetic, antifungal, anti-inflammatory, antiprotozoal, antituberculosis, and antiviral activities have been found in marine organisms and studied [2]. Many marine peptides also possess anticancer activity. Some of these peptides have been studied as cancer treatment in human clinical trials [8,9,10]. Marine anticancer peptides can kill cancer cells through different mechanisms, such as apoptosis, affecting the tubulin-microtubule imbalance, and inhibition of angiogenesis [11]. Understanding the structure and function of pharmacologically active marine-derived anticancer peptides will enhance the development of lead drug candidates.

This review focuses on the function and structure of pharmacologically active marine peptides with anticancer activity. Research on peptides derived from marine sources is underway to develop new potential cancer treatments.

## 2. Marine Organisms that are Sources of Anticancer Peptides

Many researchers have isolated anticancer peptides from various marine sources, such as, cyanobacteria, fungi, sponges, tunicates, ascidians, mollusks, and fish. Anticancer peptides cause cell death through a unique mechanism involving selectivity to cancer cells and membrane breakdown. The outer portion of the cell membrane of cancer cells has a higher concentration of anionic phosphatidylserine than found in normal cells, resulting in a total negative charge on the cell surface. As a result, the cationic anticancer peptide is attracted to the surface of the cancer cell and binds to the cell membrane, resulting in the collapse of the membrane and rapid cell death [12,13]. The structural properties of these peptides are attributable to diverse unusual amino acid residues exhibiting anticancer activity [14].

### 2.1. Cyanobacteria

Cyanobacteria (marine blue-green algae) are an abundant source of established and novel bioactive compounds with powerful therapeutic applications [15,16]. The cyanobacteria population has considerable diversity, comprising 150 genera and about 2000 species [17]. Most studies involve anticancer agents derived from marine cyanobacteria, which kill cancer cells by inducing apoptotic cell death or affecting cell signaling [5]. In addition to their cytotoxic effect on human cancer cell lines, some peptides have been found as templates for the development of new anticancer drugs. Moreover, these anticancer peptides possess anti-inflammatory and antibacterial properties [18].

### 2.2. Fungi

Marine fungi produce various secondary metabolites with biological activity and may be structurally unique [19,20]. Increasing attention regarding the pharmaceutical potential of marine fungi and screening for novel compounds with anticancer properties have led to the discovery of several related marine products. In recent years, some marine fungi-derived compounds have shown potent anticancer activity through different mechanisms, such as cell death induced via multiple cell death pathways and stimulation of cell cycle arrest [21]. Facultative marine fungi have been researched since they are known to produce novel compounds having anticancer activities [18].

### 2.3. Sponges

One of the most abundant sources of therapeutically effective products in marine organisms are marine sponges [22]. Every year, around 5300 different new compounds are being found from sponges [22]. Among the sponge-derived peptides reported so far, peptides having anticancer activity are mainly produced in *Haliclona*, *Petrosia*, and *Discodemia* [22]. Sponge-derived peptides have broad biological activities [22,23,24]. Among these peptides, dolastatins, didemnin B and aplidine have been recently reported in clinical trials [25]. Natural products isolated from sponges are of interest to the pharmaceutical industry, but most are cyclodepsipeptides, secondary metabolites which contain specific amino acids and non-amino acid moieties. For this reason, natural products isolated from sponges are difficult to isolate sufficiently in order to conduct pharmacological testing. Nevertheless, a great deal of research has been done on these products because of their various biological activities.

### 2.4. Tunicates and Ascidians

The novel structured peptides found in tunicates and ascidians often possess physiological activity. This is also the case with sack-like sea squirts that produces complex antitumor compounds. Only a small number of these are currently being used in cancer treatment [11]. Tunicates are a group of marine organisms that belong to Tunicata, a subphylum of Chordata. Although ascidians were considered as tunicates, recent studies have shown that ascidians and tunicates are actually a different species [7]. Many of these ascidian species produce anticancer peptides, steroids and antioxidants in novel structures that have biological activity [18].

### 2.5. Mollusks and Fish

Mollusks are organisms with a broad range of pharmacological uses. Mollusks are a major source of primary metabolites because they produce a variety of peptides, enzymes, polysaccharides, and lipids. In the case of a Sea hare, a shelled organism, it produces a variety of peptides in linear, cyclic and conjugated forms, with potent anticancer activity [26].

Marine fish are an important source of bioactive peptides and proteins. For this reason, the importance of fish producing novel bioactive materials is rapidly increasing. Fish-derived peptides are involved in various pharmacological effects, including antihypertensive, immunomodulatory, antioxidant, antitumor, and antimicrobial activities [18,27,28].

## 3. Bioactive Peptides with Anticancer Potential Isolated from Marine Organisms

Most of the commercially useful anticancer drugs are naturally derived compounds [25]. The suitability of novel marine-derived anticancer compounds has been determined through discovery, development, and marketing approval. These results were used for the prediction and development of potential marine-derived compounds for cancer chemotherapy [6]. Most of the marine natural products used for research are secondary metabolites. However, primary metabolites such as various types of peptides are of growing interest to the pharmaceutical industry [11].

This review describes the development of marine anticancer peptides derived from different organisms. Furthermore, a list of marine-derived anticancer peptides and their mode of action are summarized in Table 1.

### 3.1. Cyanobacteria-Derived Peptides

Cyanobacteria are regarded as a significant source of various biologically and chemically active natural peptides. Various types of peptides derived from cyanobacteria (such as linear or cyclic peptide, lipopeptides, and linear or cyclic depsipeptides) are able to induce anticancer effects on various animal and human tumor cell lines.

#### 3.1.1. Apratoxin A–D

**Figure 1:**
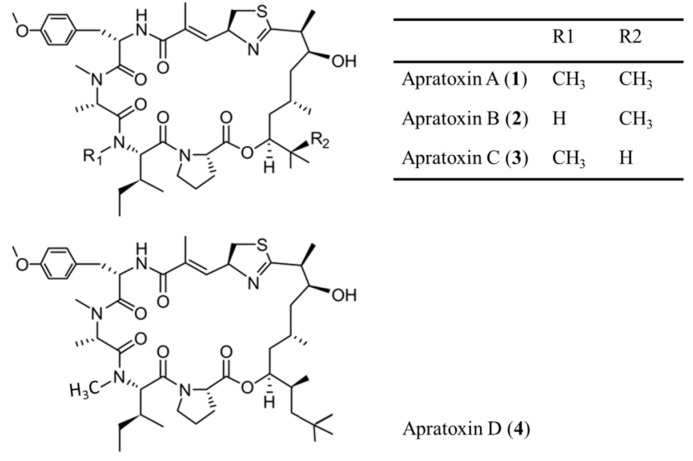
Structures of apratoxins A–D (**1**–**4**) [29,30].

Apratoxins are cyclic depsipeptides with cytotoxic activity. They were isolated from a variety of *Lyngbya* sp. found in Papua New Guinea, Palau and Guam (Figure 1) [29,30]. Apratoxins are metabolites of high cytotoxicity and have a novel skeleton composed of peptides and polyketide fragments [31]. Apratoxin A (**1**) was mainly discovered in the cyanobacterium *Lyngbya majuscula*. It showed potent cytotoxic activity in human tumor cell lines and showed half-maximal inhibitory concentration (IC_50_) values ranging from 0.36 nM and 0.52 nM in LoVo colon carcinoma cancer cells and epidermal KB carcinoma cancer cells, respectively [32]. On comparison with other natural analogs, such as apratoxin B (**2**) and C (**3**), it was observed to be structurally biologically active due to the *N*-methylated Ile residue and the hydroxyl group at C-35 [32]. In the case of apratoxin D (**4**), IC_50_ value for H-460 lung cancer cells was 2.6 nM, indicating strong in vitro cytotoxicity [33,34].

Dose-dependent DNA content analysis and gene expression profiling of apratoxin A revealed critical inhibition of cell division, delay in the G1 stage cell cycle arrest, and apoptosis-induced cell death. Apratoxin A was found to inhibit fibroblast growth factor receptor (FGFR) signaling pathway, which is generally associated with cancer [35]. Also, it prevented phosphorylation or activation of signal transducer and activator of transcription 3 (STAT3), a downstream transcriptional effector of FGFR signaling, in the functional genome approach [35]. STAT3 is expressed and activated in a broad range of cancers, which makes it an important anticancer drug target [35]. Because FGF is important for cell proliferation and angiogenesis, the inactivation of STAT3 occurs through angiogenesis mediated by the FGF signaling pathway and initiates the apoptotic cascade [35]. On the basis of proteomic studies, apratoxin A inhibits the *N*-glycosylation of endoplasmic reticulum receptors, thereby depleting cancer-associated receptor tyrosine kinases only in cancer cells [29].

#### 3.1.2. Aurilides

**Figure 2:**
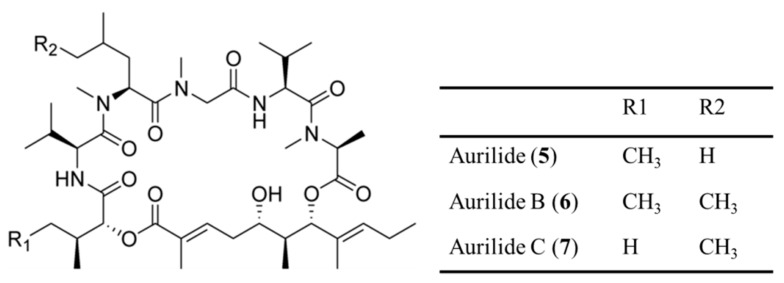
Structures of aurilide (**5**), aurilide B (**6**), and aurilide C (**7**) [36,37].

The aurilides (**5**–**7**) are cyclic depsipeptides containing a polyketoide that is part of a macrocyclic carbon skeleton and six amino acid-derived moieties (Figure 2) [36,37]. Aurilide (**5**) was isolated from the *Dolabella auricularia* in Japanese sea hare, whereas aurilide B (**6**) and C (**7**) were isolated from the oceanic cyanobacterium *Lyngbya majuscula* in the Papua New Guinea collection [36,37]. Aurilide B and C exhibited in vitro cytotoxicity against NCI-H460 (LC_50_ of 40 and 130 nM, respectively) and the neuro-2a mouse neuroblastoma cell line (LC_50_ of 10 and 50 nM, respectively) [37]. The effect of aurilide B on the NCI 60 cell line panel was also analyzed and it was found to exhibit high cytotoxicity with a 50% growth inhibition (GI_50_) value of less than 10 nM in the leukemia cell line and renal and prostate cancer cell lines [37]. The net tumor cell killing activity of aurilide B was confirmed by the National Cancer Institute (NCI)’s hollow fiber assay for preliminary in vivo screening of novel anticancer drugs.

#### 3.1.3. Bisebromoamide

**Figure 3:**
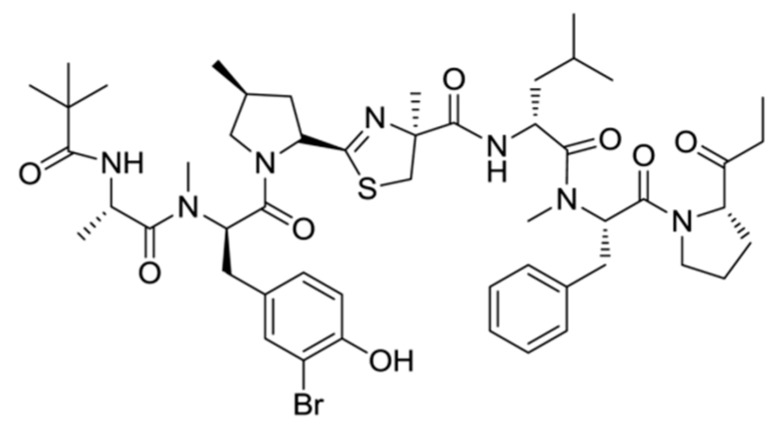
Structure of bisebromoamide (**8**) [38].

Bisebromoamide (**8**) is a linear peptide that is a marine toxic substance isolated from *Lyngbya* sp. in the Okinawan collection (Figure 3) [38]. Bisebromoamide consists of the *N*-pivaloyl-alanine, *N*-methyl-3-bromo-tyrosine, 4-methylproline, 2-(1-oxo-propyl)-pyrrolidine and 2-methylcystine, leucine, *N*-methylphenyl-alanine.

Bisebromoamide is a strong cytotoxin with an IC_50_ value of 40 ng/mL in HeLa S3 cells. Also, for a panel of 39 human cancer cell lines (JFCR39), it showed an average GI_50_ value of 40 nM. Moreover, a wealth of experimental data demonstrated that the extracellular signal-regulated protein kinase (ERK) signaling cascade recognized its targets through interaction with bisebromoamide [38].

#### 3.1.4. Coibamide A

**Figure 4:**
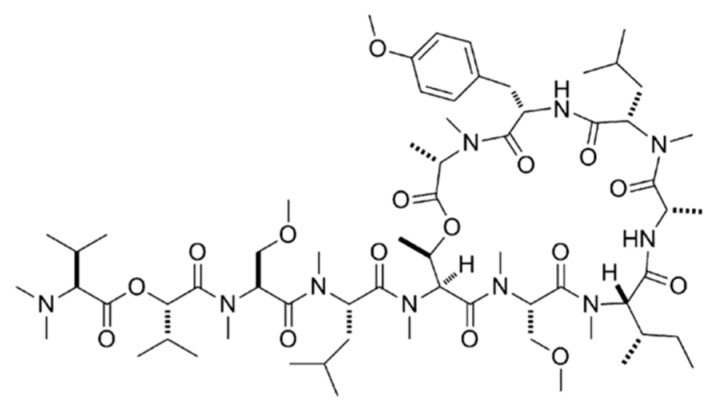
Structure of coibamide A (**9**) [39].

Coibamide A (**9**) was isolated from *Leptolyngbya* sp., a Panamanian marine cyanobacterium, which is a cyclic depsipeptide (Figure 4) [39]. Testing of coibamide A in the NCI-H460 lung cancer cells and mouse neuro-2a cells showed strong cytotoxicity with LC_50_ values of less than 23 nM. Furthermore, coibamide A, a powerful cancer cell toxin with an unique selectivity for the NCI 60 cancer cell line panel, showed significant cytotoxicity against HL-60 human myeloid cells, LOX IMVI human melanoma cell, MDA-MB-231 breast cancer cells and SNB-75 cells with low nM potency. In addition, powerful anti-proliferative activity of the cancer cell was found via a novel target or mechanism of action using COMPARE assays [39].

#### 3.1.5. Cryptophycin

**Figure 5:**
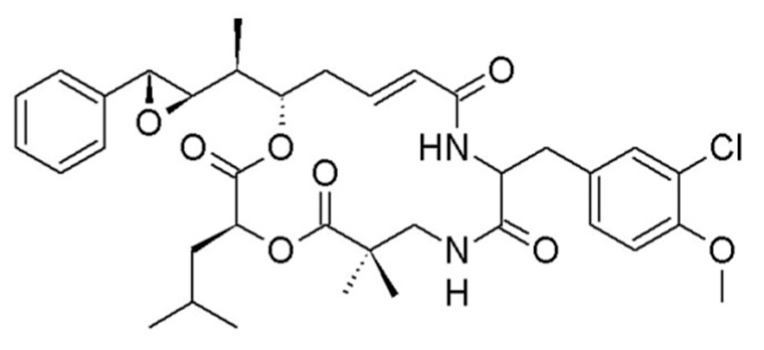
Cryptophycin (**10**) isolated from the cyanobacterium *Nostoc* sp. [35].

Cryptophycin (**10**) was isolated from the marine cyanobacteria *Nostoc* sp. ATCC 53789 and GSV 224 and is a depsipeptide with potent antifungal activity (Figure 5) [40]. Cryptophycin bound strongly to the microtubule ends at the vinca-binding site and inhibited the microtubule polymerization. It showed marked cytotoxicity with an IC_50_ value of less than 50 pM for multidrug-resistant (MDR) tumor cell lines [41].

The synthetic derivative of cryptophycin, cryptophycin-52 (LY355703), is produced by total synthesis. Cryptophycin-52 induced apoptosis, which is confirmed through the hyperphosphorylation of Bcl-2, cell cycle arrest, and growth inhibition in preclinical trials for the in vitro human non-small cell lung carcinoma (NSCLC) cell line [42]. A clinical phase II study of cryptophycin-52 revealed the antitumor effect in advanced NSCLC and platinum-resistant advanced ovarian cancer in patients [43,44].

#### 3.1.6. Desmethoxymajusculamide C

**Figure 6:**
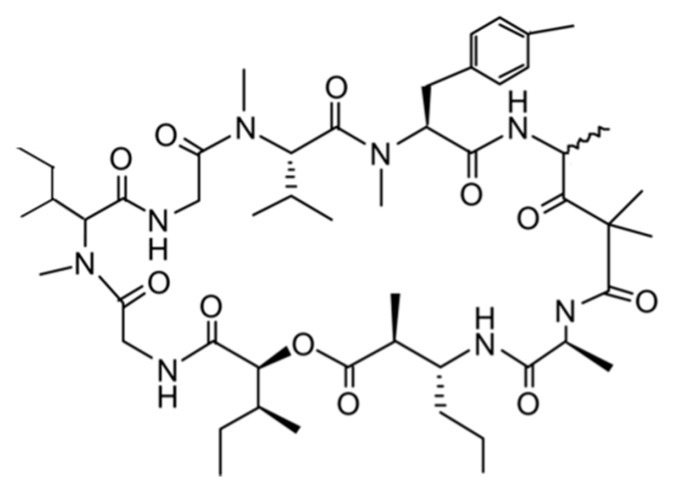
Structure of desmethoxymajusculamide C (**11**) [45].

Desmethoxymajusculamide C (**11**) is a cyclic depsipeptide produced by the *L. majuscula*. It has strong and selective antitumor activity when tested against HCT-116 human colon carcinoma cells as demonstrated by an IC_50_ of 20 nM and the destruction of cell microfibrils networks (Figure 6) [45]. In the disk diffusion assay, linear desmethoxymajusculamide C maintained powerful actin depolymerization ability as well as solid tumor selectivity [45].

#### 3.1.7. Grassypeptolides

**Figure 7:**
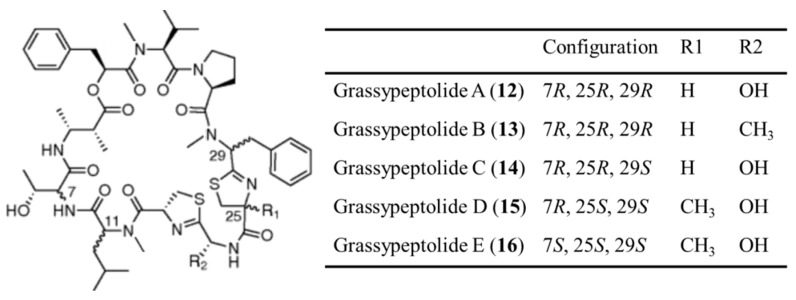
Structures of grassypeptolide A–E (**12**–**16**) [46,47].

Grassypeptolides A–C (**12**–**16**) are cyclic depsipeptides containing bis-thiazoline and were isolated from *Lyngbya confervoides* [46]. Grassypeptolides also contains an unusual β-amino acid (2-methyl-3-aminobutyric acid), many d-amino acids, and a number of *N*-methylated amino acids. When the ethyl substituent of Grassypeptolide A (**12**) (IC_50_ = 1.22 and 1.01 μM against HT29 and HeLa cancer cell lines, respectively) turned to the methyl group in grassypeptolide B (**13**), the activity decreases to an extent (3–4-fold; IC_50_ = 4.97 and 2.93 μM), while the reversal of the Phe unit adjacent to the bis-thiazoline moiety (grassypeptolide C (**14**)) results in 16–23 fold greater efficacy (IC_50_ = 76.7 and 44.6 nM) (Figure 7) [46]. Grassypeptolide A and C induce G1 cell cycle arrest at lower concentrations and induce G2/M cell-cycle arrest at higher concentrations in HeLa cells [46].

Grassypeptolide D (**15**) and E (**16**) exhibit potent cytotoxicity against HeLa (IC_50_ = 335 and 192 nM, respectively) and mouse neuro-2a blastoma cells (IC_50_ = 599 and 407 nM, respectively) (Figure 7) [47].

#### 3.1.8. Hantupeptin A

**Figure 8:**
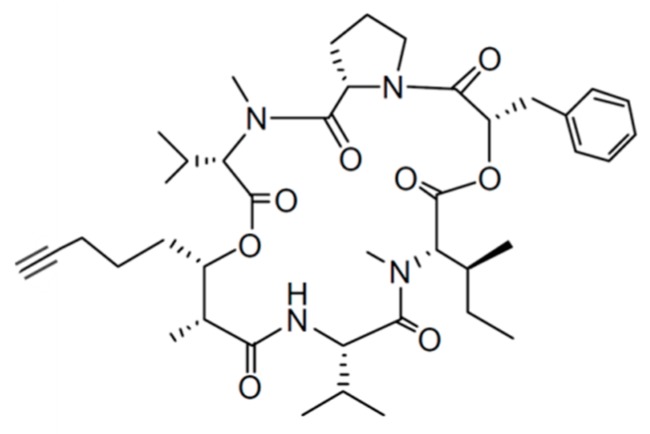
Hantupeptin A (**17**) isolated from cyanobacterium *Lyngbya majuscula* [48].

Hantupeptin A (**17**) is a cyclodepsipeptide with a potent cytotoxicity, isolated from *Lyngbya majuscula* (Figure 8) [48]. Hantupeptin A is a 19-membered cyclic tetrapeptide, which consists of α-amino acids, α-hydroxy acid residue (either phenyl lactic acid, proline, *N*-methylvaline, valine, or *N*-methylisoleucine), and an α-methyl-β-hydroxy acid unit with an alkyne at the C-terminal end. The hydroxyl group is attached on the carbon (C-35) of an unusual hydroxy acid, 3-hydroxy-2-methyloctynoic acid [49,50]. Hantupeptin A has shown cytotoxicity with IC_50_ values of 32 nM and 4.0 μM when tested against MOLT-4 leukemia and MCF-7 breast cancer cells, respectively [49,50].

#### 3.1.9. Hectochlorin

**Figure 9:**
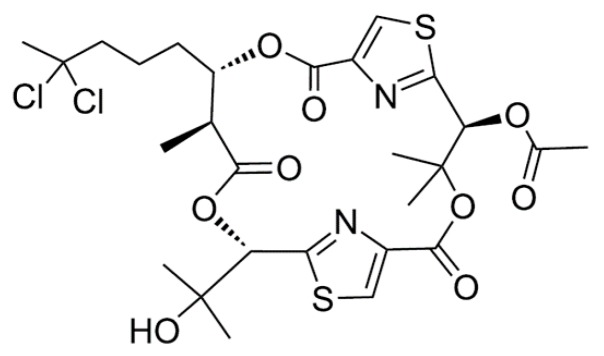
Structure of hectochlorin (**18**) [51].

Hectochlorin (**18**) was identified from *L. majuscula* and showed potent activity in promoting actin polymerization (Figure 9) [51].

The pharmacological target for hectochlorin has been proposed as actin microfilaments owing to the cumulation of CA46 cells in the G2/M phase. Subsequently, hectochlorin induced actin polymerization with a half-maximal effective concentration (EC_50_) value of 20 μM in PtK2 cells. Among the NCI 60 cancer cell lines, 23 cancer cell lines such as colon melanoma, ovarian tumor, and renal cells showed strong cytotoxicity. Hectochlorin (**18**) showed cytotoxic activity with IC_50_ values of 20 and 300 nM for CA46 human Burkitt lymphoma cell and PtK2 cells, respectively [51]. However, the dose-response curve of hectochlorin was flat, suggesting that hectochlorin is more antiproliferative than cytotoxic [51].

#### 3.1.10. Hormothamnin A

**Figure 10:**
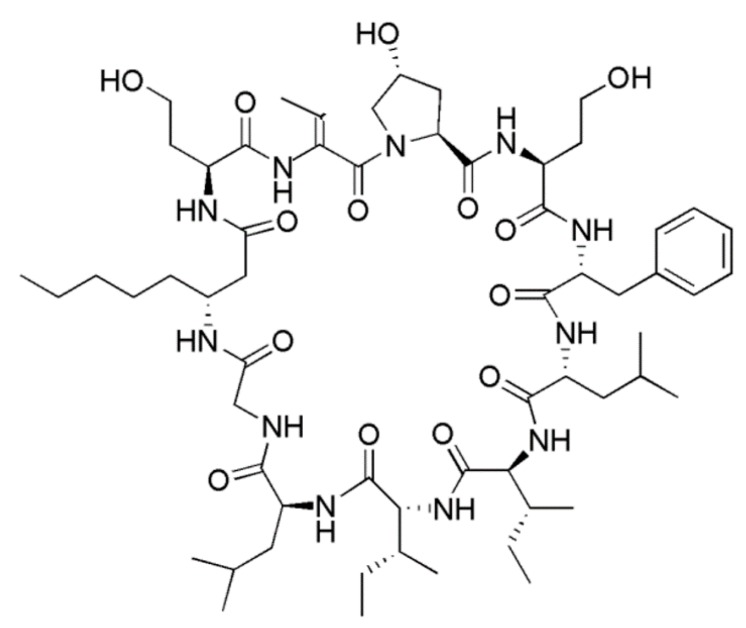
Hormothamnin A (**19**) isolated from the cyanobacterium *Hormothamnion enteromorphoides* [52].

Hormothamnin A (**19**) isolated from *Hormothamnion enteromorphoides* is a cyclic undecapeptide containing six common and five uncommon or novel amino acid residues (Figure 10) [52]. Hormothamnin A (**19**) was significantly cytotoxic in a variety of solid cancer cell lines, including human lung cell (SW1271 and A529), murine melanoma cell (B16-F10), and human colon cell (HCT-116) [47] with IC_50_ values ranging from 0.13 to 0.72 μg/mL.

#### 3.1.11. Itralamide A and B

**Figure 11:**
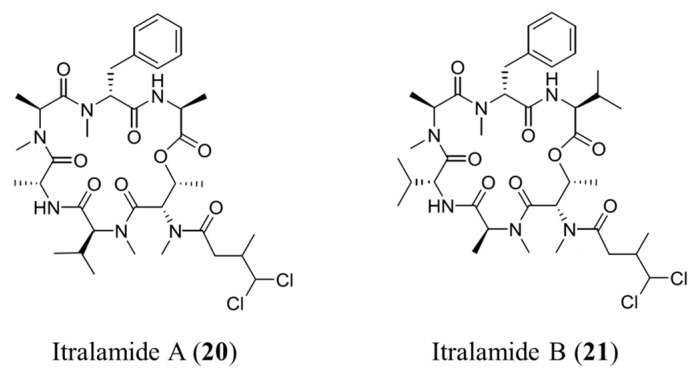
Itralamide A (**20**) and B (**21**) isolated from cyanobacterium *Lyngbya majuscula* [53].

Caribbean sources of *L. majuscula* yielded two cytotoxic cyclodepsipeptides: itralamide A (**20**) and B (**21**) (Figure 11) [53]. Itralamide B (**21**) showed a higher activity than the former, in addition to displaying moderate activity against human embryonic kidney cells 293 (HEK293, IC_50_ = 6 μM). Itralamide A (**20**) is structurally similar to itralamide B, but has a much lower activity [53].

#### 3.1.12. Lagunamides

**Figure 12:**
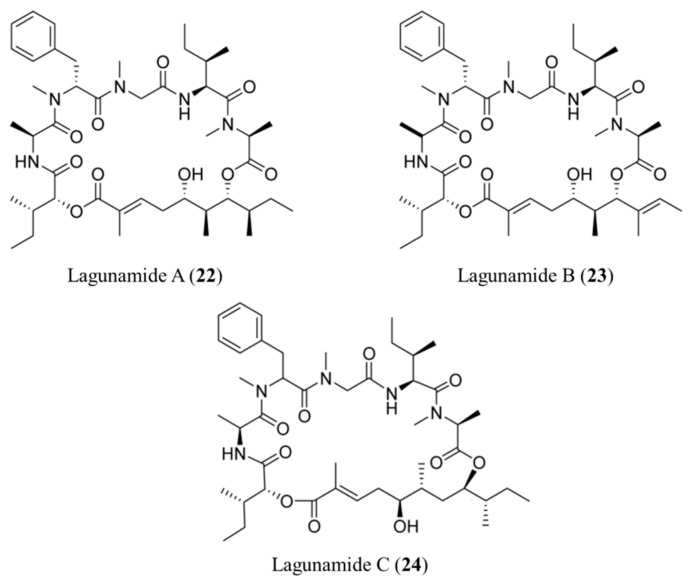
Structures of Lagunamide A (**22**), B (**23**), and C (**24**) [54,55].

The cyclodepsipeptides lagunamide A (**22**) and B (**23**) were isolated from the filamentous marine cyanobacterium *L. majuscule*. These compounds were cytotoxic against P388 murine leukemia cell lines with IC_50_ values of 6.4–20.5 nM, respectively (Figure 12) [54]. Furthermore, a biochemical analysis using HCT8 and MCF7 cancer cells indicated that lagunamide A and B exhibit cytotoxicity by inducing mitochondria-mediated apoptosis [54].

Lagunamide C (**24**) exhibited strong cytotoxicity against P388, A549, PC3, HCT8, and SK-OV3 cell lines with IC_50_ values ranging from 2.1–24.4 nM [55].

#### 3.1.13. Largazole

**Figure 13:**
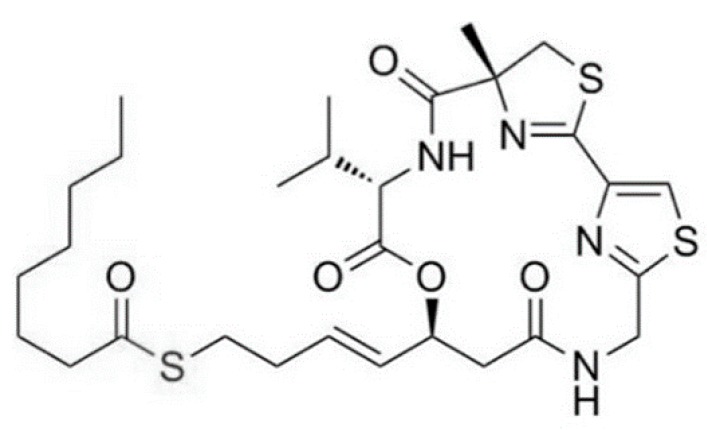
Largazole (**25**) isolated from cyanobacterium *Symploca* sp. [56].

Largazole (**25**) is a cytotoxic cyclic depsipeptide isolated from *Symploca* sp. (Figure 13) [56]. Largazole is a potent growth inhibitor against transformed human mammary epithelial cells (MDA-MB-231, GI_50_ of 7.7 nM) over non-transformed murine mammary epithelial cells (NMuMG, GI_50_ of 122 nM) [56]. These molecules also showed strong antiproliferative activity of the transformed U2OS fibroblastic osteosarcoma cells (GI_50_ = 55 nM). However, it was less active in non-transformed NIH3T3 fibroblasts (GI_50_ = 480 nM) than in transformed U2OS cells (GI_50_ = 55 nM). Due to its biological activity and selectivity, several research groups investigated the total chemical synthesis of largazole [57,58,59,60].

#### 3.1.14. Laxaphycin A and B

**Figure 14:**
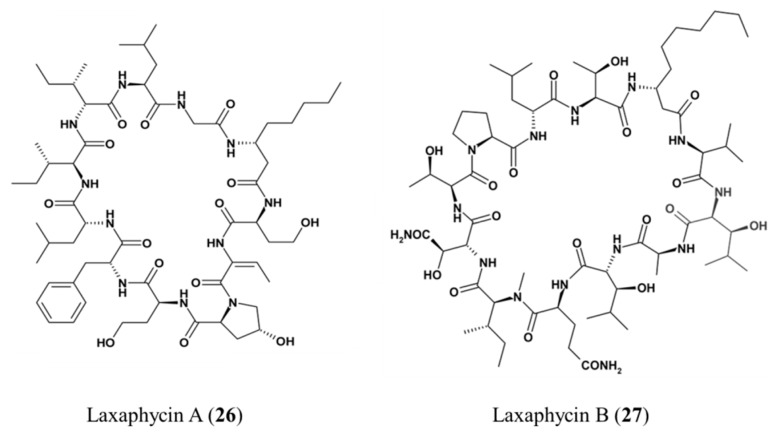
Structures of laxaphycin A (**26**) and laxaphycin B (**27**) [61,62].

Laxaphycins (**26**, **27**) are cyclic peptides isolated from *Anabaena laxa.* Laxaphycins A (**26**) and B (**27**) were re-isolated from an assemblage of *L. majuscule*, together with *Anabaena* sp. [61,62]. Laxaphycin A is a cyclic undecapeptide containing a 3-aminooctanoic acid residue, and laxaphycin B has a 3-aminodecanoid acid moiety (Figure 14). Laxaphycin A exhibited weak cytotoxicity on a panel of solid cancer cell lines. These include A549 (human lung adenocarcinoma epithelial cell), MCF7 (human breast adenocarcinoma cell), PA1 (human ovarian teratocarcinoma cell), PC3 (human prostate cancer cell), DLD1 (human colorectal adenocarcinoma cell), and M4Beu (human pigmented malignant melanoma) cell lines [63]. Laxaphycins A and B also showed synergistic inhibitory effects on cancer cells in an antifungal assay [63]. Furthermore, laxaphycin B exhibited strong anticancer activity against both sensitive and resistant cancer cell lines [63].

The mode of action of laxaphycin B (**27**) alone may be different from that of laxaphycin A in combination with laxaphycin B (**26**) [63]. Therefore, laxaphycins A and B could offer new visions into the clinical use of combinatorial drugs in the treatment of cancer.

#### 3.1.15. Lyngbyabellin A, E, and B

**Figure 15:**
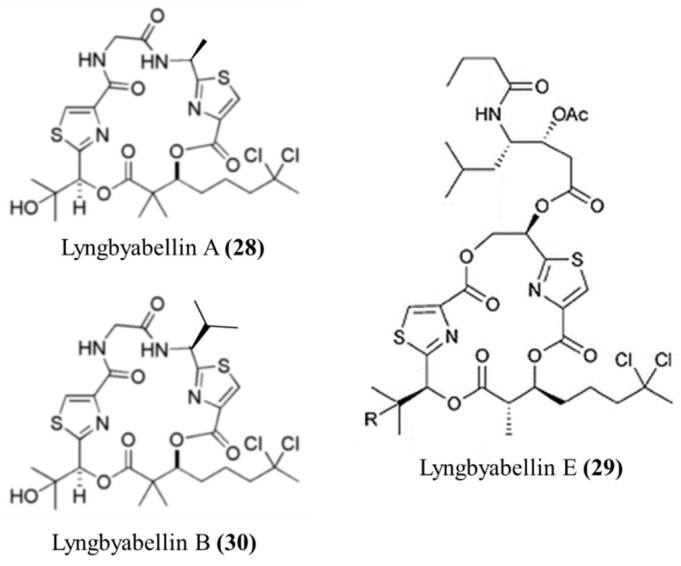
Structures of Lyngbyabellin A (**28**), E, (**29**) and B (**30**) [64,65].

Lyngbyabellin A (**28**) and E (**29**) isolated from *L. majuscula* are hectochlorin-related lipopeptides with potent actin polymerization activity, and lyngbyabellin A (**28**) showed moderate cytotoxicity in KB (IC50 values of 0.03 μg/mL) and LoVo (IC50 = 0.5 μg/mL) cells (Figure 15) [64,65]. It disrupted the cellular actin microfilament network in A10 cells at 0.01–7.2 μM. Furthermore, lyngbyabellin A was fatal to mice when injected at a median lethal dose (LD_50_) value of 1.2–1.5 mg/kg [64].

Lyngbyabellin E (**29**) also exhibited actin polymerization ability at 60 nM; it completely blocked the cellular microfilaments in A10 cells, forming binucleated cells [65]. Furthermore, it showed cytotoxic activity against NCI-H460 (human lung carcinoma cell, LC50 values of 0.4 μM) and neuro-2a (mouse neuroblastoma cell (LC50 = 1.2 μM) [65].

Lyngbyabellin B (**30**) showed effects on A10 human Burkitt lymphoma cells [64]. Furthermore, the cytotoxic effect of lyngbyabellin B was weaker than that of lyngbyabellin A against KB (IC50 values of 0.1 μg/mL) and LoVo (IC50 = 0.83 μg/mL) cells [64].

#### 3.1.16. Lyngbyastatin 4–7

**Figure 16:**
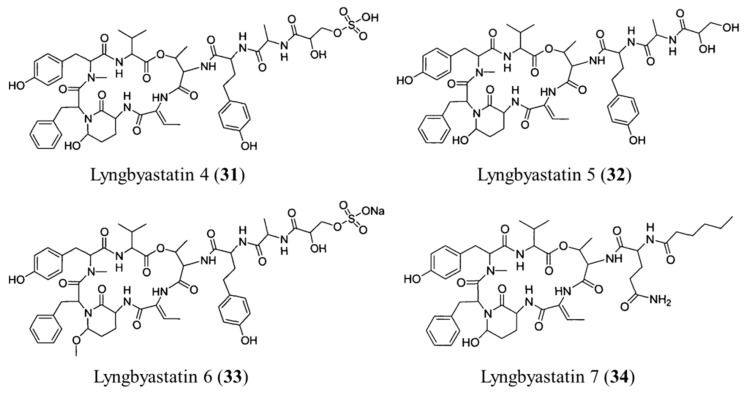
Structures of lyngbyastatin 4–7 (**31**–**34**) [67].

Many cyclic depsipeptides isolated from marine cyanobacteria show strong inhibition of serine proteases such as pepsin, trypsin, α-chymotrypsin, and elastase. Serine protease is involved in a variety of disease states, including extermination of the extracellular surface of the cartilage covering bones (articular cartilage) in arthritic refractory, emphysema, and inflammatory infections. For this reason, inhibition of serine proteases will be available as a new potential drug target for cancer therapy [66]. Lyngbyastatin 4–7 were depsipeptides isolated from *Lyngbya* (**31**–**34**) (Figure 16) [67].

Lyngbyastatins showed in vitro inhibitory effect against elastase, chymotrypsin, and trypsin. Lyngbyastatins 4–7 are potent inhibitors of elastase with IC_50_ values between 120 and 210 nM [67].

#### 3.1.17. Symplocamide A

**Figure 17:**
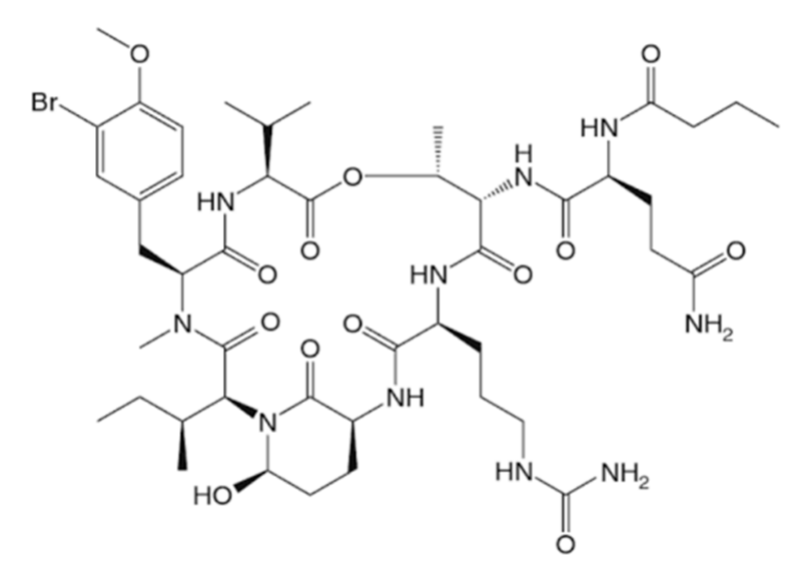
Structure of Symplocamide A (**35**) [68].

Symplocamide A (**35**), isolated from *Symploca* sp. is a cyclodepsipeptide that showed potent cytotoxicity to non-small cell lung cancer cells H-460 (IC_50_ = 40 nM) and to neuro-2a neuroblastoma cells (IC_50_ = 29 nM) (Figure 17) [68]. Symplocamide A also inhibited serine proteases, and its inhibitory activity on chymotrypsin was 200-fold higher than trypsin (IC_50_ = 0.38 μM) [68].

#### 3.1.18. Tasiamides

**Figure 18:**
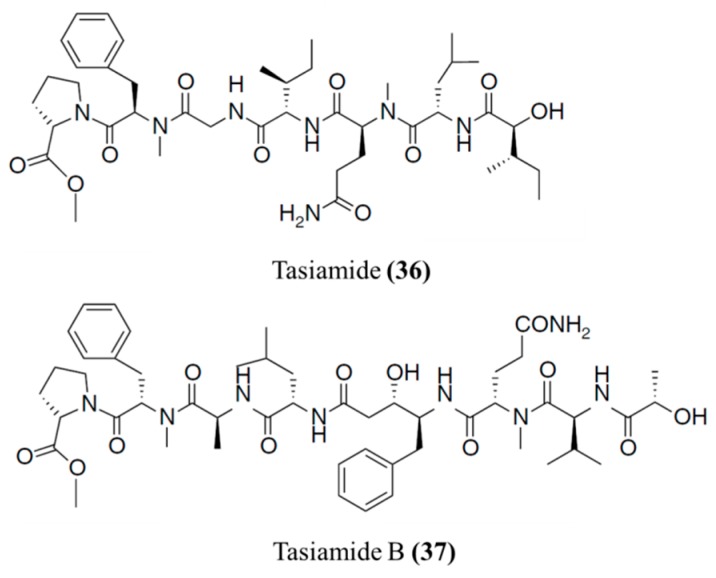
Structures of tasiamide (**36**) and tasiamide B (**37**) [69,70,71].

Tasiamide (**36**) and tasiamide B (**37**) are linear peptides isolated from the cyanobacterium *Symploca* sp. (Figure 18) [69]. Tasiamide (**36**) showed strong toxicity to human nasopharyngeal carcinoma (KB) and human colon carcinoma (LoVo) cells, with IC_50_ values of 0.48 and 3.47 μg/mL, respectively [69].

Tasiamide B (**37**) (an octapeptide) contains 4-amino-3-hydroxy-5-phenylpentanoic acid, a unique amino acid moiety, and have cytotoxity against KB cell lines [70]. The synthetic analogues of tasiamide also inhibited KB and non-small cell lung tumor cell lines [71].

#### 3.1.19. Veraguamide A, D, and E

**Figure 19:**
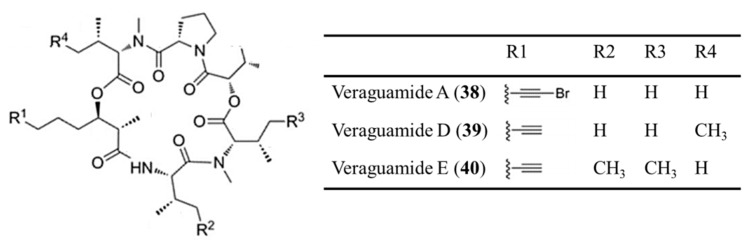
Veraguamide A (**38**), D (**39**), and E (**40**), isolated from *Oscillatoria margaritifera* [72,73].

Veraguamides, a family of cyclodepsipeptides, were isolated from *Oscillatoria margaritifera* (Figure 19) [72]. Veraguamide A (**38**) was highly toxic in the H-460 human lung cancer cell line (LD_50_ = 141 nM) [72]. In addition, veraguamides A–G were isolated from *Symploca* cf. *hydnoides*. Veraguamides D (**39**) and E (**40**) showed cytotoxicity against both HT 29 and HeLa cell lines (IC_50_ = 0.5–1.5 μM) [73]. The increase in the hydrophobicity of specific units in veraguamides results in their increased activity.

### 3.2. Fungi-Derived Peptides

Marine fungi produce biologically active and chemically solitary peptides that have offered research opportunities for effective cancer treatments. However, fungi-derived peptides, like sponge-derived peptides, are mostly secondary metabolites that are difficult to isolate. For this reason, the fungi-derived peptides are less studied than other marine peptides [11].

#### 3.2.1. Azonazine

**Figure 20:**
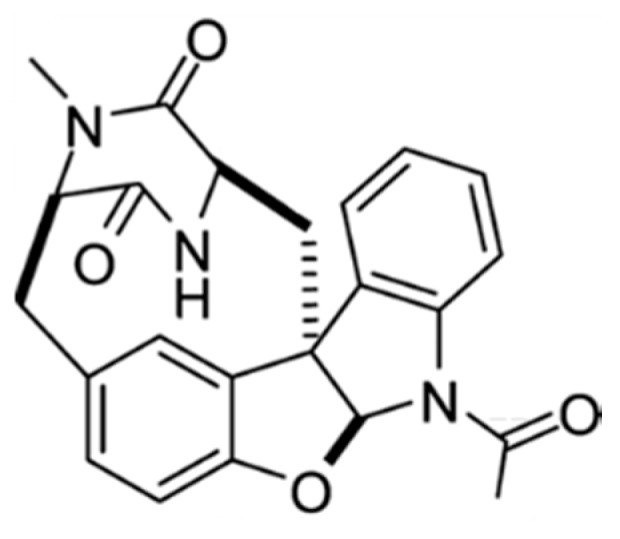
Azonazine (**41**) isolated from *Aspergillus insulicola* [74].

Azonazine (**41**) is a hexacyclic dipeptide from *Aspergillus insulicola*, a Hawaiian marine sediment-derived fungus (Figure 20) [74]. Azonazine showed cytotoxicity with IC_50_ values < 15 ng/mL against HCT-116. This peptide showed anti-inflammatory activity by inhibition of NF-*κ*B luciferase (IC_50_ = 8.37 μM) and nitrite production (IC_50_ = 13.70 μM).

#### 3.2.2. Sansalvamide A

**Figure 21:**
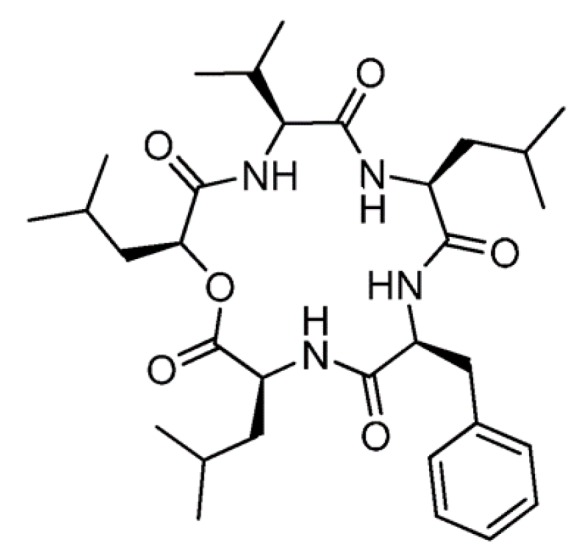
Structure of sansalvamide A (**42**) [75].

The cyclic depsipeptide, Sansalvamide A (**42**), was isolated from *Fusarium* sp. living on the marine plant *Halodule wrightii* (Figure 21) [75]. This peptide showed cytotoxicity against different cell lines such as pancreatic, colon, breast, prostate sarcoma and melanoma cancer cell lines, which suggests it is a potent chemotherapeutic agent (cytotoxicity against the HT29 cell was IC_50_ = 4.5 μg/mL) [76]. Until now, the exact mechanism of sansalvamide A is unknown. A recent research showed interaction between the HSP90 heat shock protein and client cancer proteins in mammalian cell lines. Sansalvamide A-amid is a synthetic peptide that has similar action to Sansalvamide A [76]. Sansalvamide A-amid was reported to bind to the *N*-middle domain of HSP90 and allosterically inhibits the formation of protein complex needed to promote tumor growth [76]. In addition, Sansalvamide A-amid caused G1 phase cell cycle arrest in the AsPC-1 and CD18 human pancreatic cancer cell lines. Ssansalvamide A acts as an inhibitor of topoisomerase I by inducing cell death by negligible apoptosis in several cancer cell lines [76].

#### 3.2.3. Scopularide A and B

**Figure 22:**
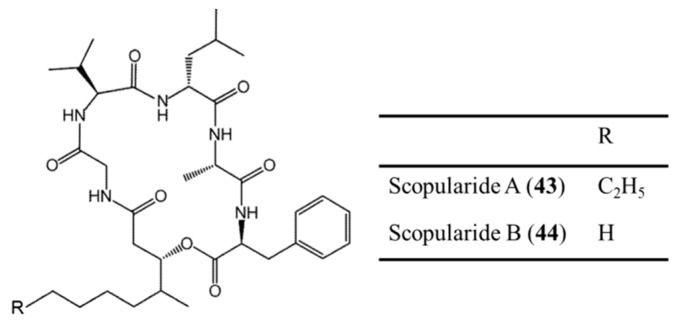
Scopularide A (**43**) and B (**44**) isolated from fungi *Scopulariopsis brevicaulis* [77].

Scopularide A (**43**) and B (**44**) are cyclodepsipeptides isolated from the marine fungi *Scopulariopsis brevicaulis* and the marine sponge *Tethya aurantium* (Figure 22) [77]. Both compounds have the ability to inhibit the growth of pancreatic and colon tumor cell lines [77]. These peptides have no antimicrobial activity against gram-negative bacteria and have weak activity against gram-positive bacteria [77]. However, the cytotoxicity of various tumor cell lines including the Colo357 and Panc89 pancreatic tumor cell lines and the HT29 colon tumor cell line was confirmed at the concentration of 10 μg/mL (IC_50_) [77]. 

### 3.3. Sponge-Derived Peptides

Anticancer peptides from sponges are mostly cyclodepsipeptides with unusual amino acids or non-amino acid parts. These sponge-derived peptides showed a broad range of anticancer or antitumor activity [78].

#### 3.3.1. Arenastatin A

**Figure 23:**
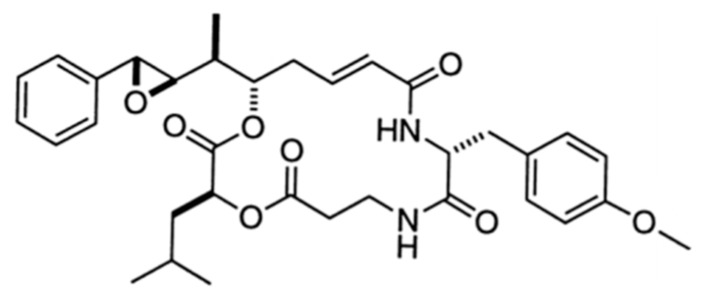
Structure of arenastatin A (**45**) [79].

Arenastatin A (**45**), a macrocyclic depsipeptide, was isolated from *Dysidea arenaria* with extremely strong cytotoxic activity (IC_50_ = 5 pg/mL) against a human nasopharyngeal carcinoma (KB) cell line (Figure 23) [79]. Arenastatin A inhibits microtubule assembly due to the binding rhizoxin/maytansine sites of tubulin, resulting in cytotoxicity [80,81]. However, arenastatin A is unstable in mouse serum; there is little in vivo antitumor activity. The high sensitivity of the ester linkage to hydrolysis and degradation in blood is a disadvantage [82,83]. The synthesized 15-*tert*-butyl derivative of arenastatin A overcomes this disadvantage and exhibits improved serum stability as well as in vivo antitumor activity.

#### 3.3.2. Discodermin A–H

Discodermins are cytotoxic tetradecapeptides obtained from *Discodermia* sp. containing a macrocyclic ring formed by lactonization of a threonine amino acid unit at the C-terminal and 13–14 common and unusal amino acids-linked structures [84].

**Figure 24:**
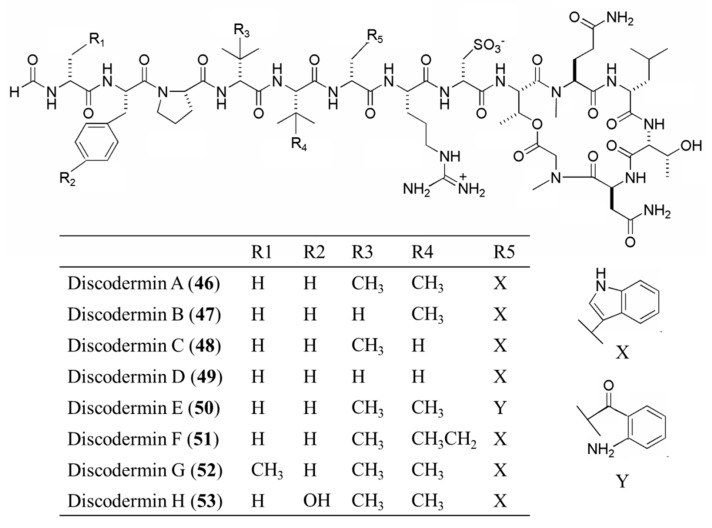
Structure of discodermin A–H (**46**–**53**) [84].

Discodermin A–H (**46**–**53**) are all cytotoxic; tests against murine leukemia cell line (P388), human lung cell line (A549), and the inhibition of starfish development resulted in IC_50_ values of 0.02 to 20 μg/mL [84]. Among these, discodermin A (**46**) was shown to permeabilize the plasma membrane of tissues and vascular smooth muscle cells (Figure 24) [84].

#### 3.3.3. Geodiamolide H

**Figure 25:**
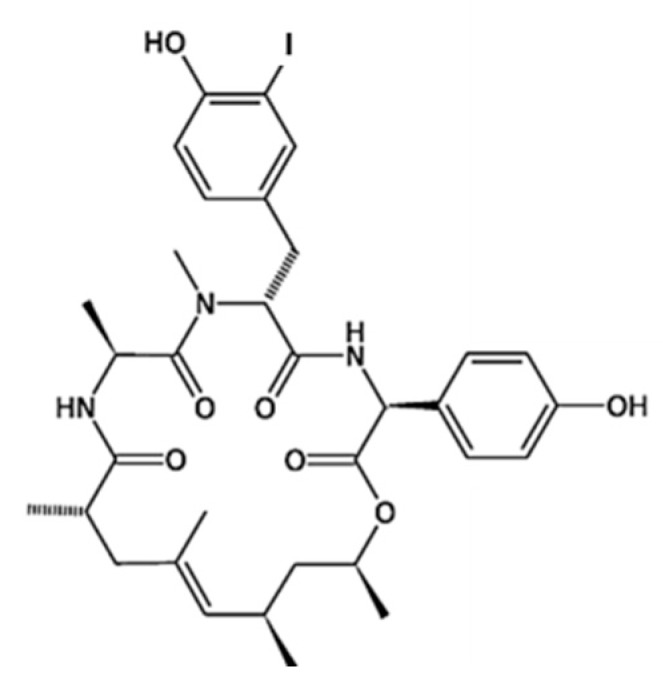
Geodiamolide H (**54**) isolated from *Discodermia* sp. [85,86,87].

Geodiamolides were originally isolated from *Geodia* sp. as cyclic forms of peptides consisting of three amino acid residues with a common polyketide unit [85,86]. Geodiamolide H (**54**) is a cyclic depsipeptide isolated from *G. corticostylifera*, which showed in vitro cytotoxicity against several human cancer cell lines such as ovarian cancer, OV Car-4 (G_100_ = 18.6 nM). Also, it has antiproliferative activity against the human breast cancer cells by altering the actin cytoskeleton (Figure 25) [87].

#### 3.3.4. Hemiasterlin

**Figure 26:**
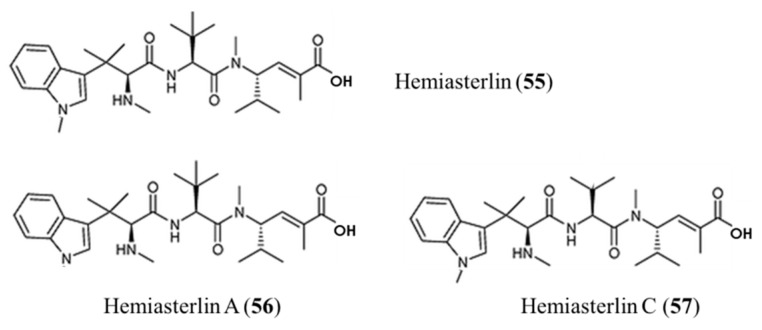
Hemiasterlin (**55**), hemiasterlin A (**56**), and hemiasterlin C (**57**) isolated from the marine sponge *Hemiasterella minor* [88,89,90].

Hemiasterlin (**55**), a linear tripeptide consisting of three sterically congested amino acids, was isolated from *Hemiasterella minor* [88]. In particular, Hemiasterlin, hemiasterlin A and hemiasterlin C (**55**–**57**) showed strong cytotoxic activity against the P388 leukemia cell (Figure 26) [88,89,90]. Hemiasterlins show inhibition with IC_50_ ranges of 0.0484–0.269 nM and 0.404–10.3 nM in PC3 and NFF cells, respectively. They block mitotic spindle formation and induce mitotic arrest and apoptosis, resulting in tubulin depolymerization by binding to the Vinca alkaloid binding site [91]. Hemiasterlin is in phase I clinical trials for patients with solid malignancies [92].

HT1286 (also called SPA-110 or taltobulin), a synthetic derivative of hemiasterlin, showed stronger cytotoxicity against human cancer cell lines than hemiasterlin, although both hemiasterlin and HT1286 have a similar mechanism of action. HT1286 has been shown to inhibit the growth of human tumor xenografts in mice, in a preclinical study [93]. The observed side effects, including alopecia, pain, and nausea, resulted in the cessation of HT1286 phase I clinical trials. However, HT1286 has high potential in the treatment of solid tumors in humans. Therefore, the development of new synthetic derivatives with fewer side effects is an important step toward the use of hemiasterlin as an anticancer drug [94,95].

#### 3.3.5. Homophymine A–E and A1–E1

**Figure 27:**
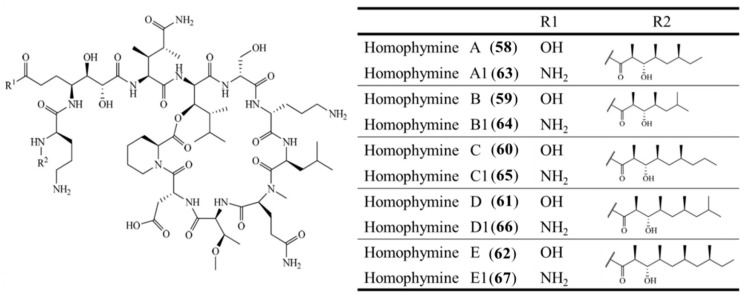
Structures of homophymine A–E (**58**–**62**) and A1–E1 (**63**–**67**) [96].

Homophymine A–E (**58**–**62**) and A1–E1 (**63**–**67**), isolated from *Homophymia* sp. are cyclodepsipeptides with very strong cytotoxicity against tumor cell lines (Figure 27) [96]. Homophymines (**58**–**67**) showed strong cytotoxicity with IC_50_ values of 2–100 nM against a panel of human cancer cell lines. They exhibited the highest activity against human prostate adenocarcinoma (PC3) and ovarian adenocarcinoma (SK-OV3) cell lines. Additional research on homophymines (**58**–**67**) was conducted to determine whether these were toxic or antiproliferative. They have been shown to induce apoptosis on the part of a caspase-independent mechanism and cytotoxicity through non-specific mechanism [96]. Homophymine A1–E1 (**63**–**67**) differ from homophymine A–E (**58**–**62**) owing to an amide residue instead of the carboxylic acid in the 4-amino-2,3-dihydroxy-1,7-heptanedioic acid residue and exert stronger potency than their corresponding homophymine A–E structures (**58**–**62**) [96].

#### 3.3.6. Jaspamide

**Figure 28:**
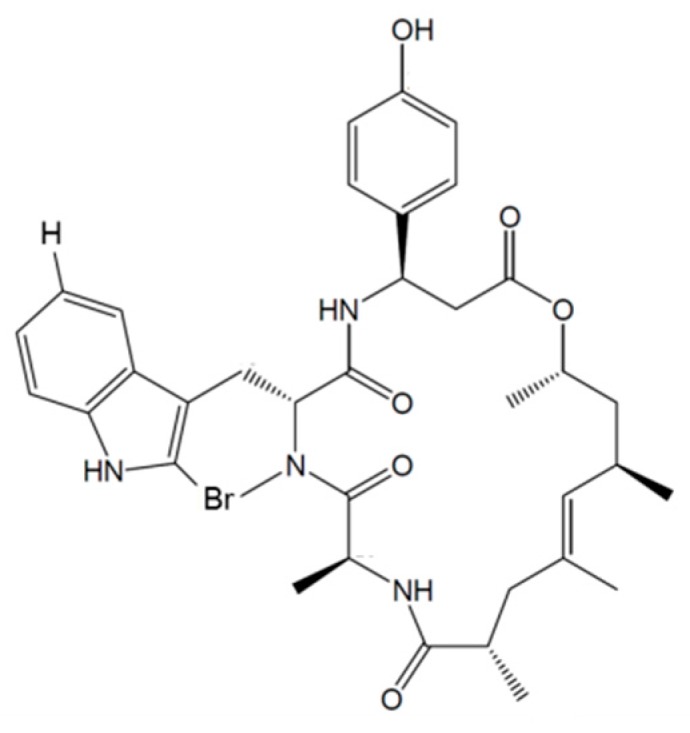
Structure of jaspamide (**68**) [97].

Jaspamide (Jasplakinolide, **68**) was isolated from *Jaspis johnstoni* as a cyclic depsipeptide with a 15-carbon macrocyclic ring of three amino acid residues (Figure 28) [97].

Jaspamide was found to be highly cytotoxic with an IC_50_ value of 0.04 ng/mL in P388 murine leukemia cells. It is a bioactive peptide that induces apoptosis in human leukemia cell lines and brain tumor Jurkat T cells by activation of caspase-3 protein expression and decrease in Bcl-2 levels [97,98,99].

In particular, transformed cell lines were more sensitive to jaspamide-induced cell death (apoptosis) than normal non-transformed cells [98].

Apoptosis induced by Jaspamide was associated with caspase-3 activation, decreased Bcl-2 protein expression, and increased Bax levels, suggesting that jaspamide induced a caspase-independent cell death pathway for cytosolic and membrane changes in apoptosis cells, and a caspase-dependent cell death pathway for PARP protein degradation [99].

#### 3.3.7. Koshikamide B and F–H

**Figure 29:**
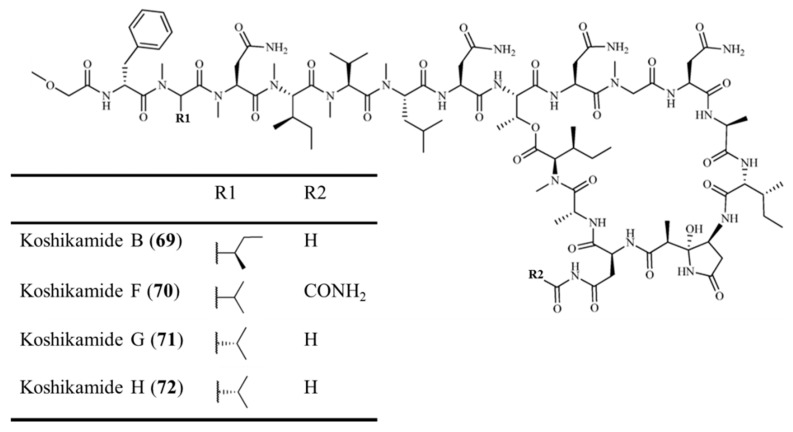
Structures of koshikamide B (**69**) and F–H (**70**–**72**) [100,101].

Koshikamide B (**69**) has been identified as the major cytotoxic constituent in two separate collections of the *Theonella* sp. Koshikamide B is a peptide lactone of 17-residue consisting of 6 proteinogenic amino acids, 2 D-isomers of proteinogenic amino acids, 2 unusual amino acids, and 7 *N*-methylated amino acids (Figure 29) [100]. It was confirmed that the IC_50_ values for human leukemia cells (P388) and human colon tumor (HCT-116) cell lines were 0.22 and 3.7 μM, respectively [100].

Koshikamide F–H (**70**–**72**) are 17-residue depsipeptides with macrolactone, their IC_50_ values were inhibited entry from 2.3 to 5.5 μM. (Figure 29) [101]. Koshikamide H (**72**) was found to have moderate cytotoxicity against the HCT-116 colon cancer cell line with IC_50_ value of 10 μM. However, koshikamides F–H did not inhibit the growth of *Candida albicans*, indicating that it is cytotoxic without antifungal activity [101].

#### 3.3.8. Microcionamide A and B

**Figure 30:**
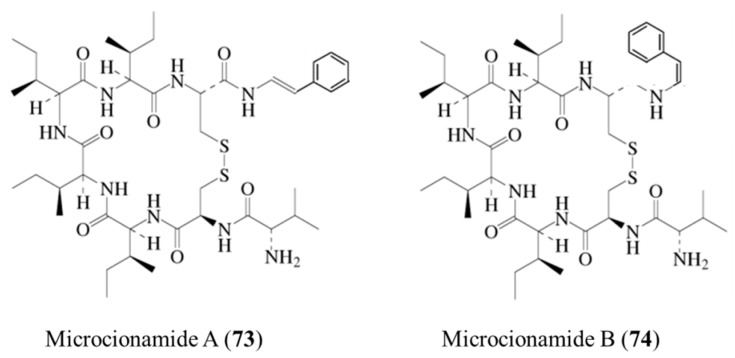
Structures of microcionamide A (**73**) and B (**74**) [102].

Microcionamide A (**73**) and B (**74**) isolated from the *Clathria* (*Thalysias*) *abietina* are linear peptides cyclized via cyclic hexapeptides. Microcionamide A (A) and B (B) showed significant cytotoxicity with IC_50_ ranges of 125 (A) and 177 nM (B) and 98 (A) and 172 nM (B) in MCF-7 and SKBR-3 cells, respectively (Figure 30) [102]. In addition, these compounds were found to induce apoptosis in MCF-7 cells within 24 h at 5.7 μM. Microcionamides A and B showed cytotoxicity for *Mycobacterium tuberculosis* H_37_Ra with MIC values of 5.7 μM [102].

#### 3.3.9. Orbiculamide A

**Figure 31:**
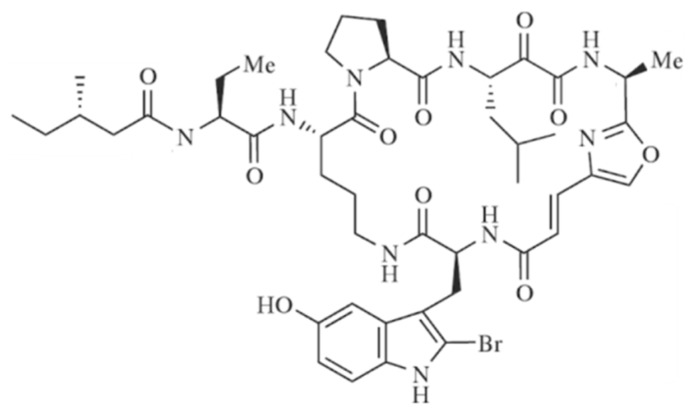
Structure of orbiculamide A (**75**) [103].

Orbiculamide A (**75**) is a cyclic peptide from *Theonella* sp. with reported cytotoxicity in P388 murine leukemia cells (IC50 = 4.7 μg/mL) and several other melanoma cell lines (Figure 31) [103].

#### 3.3.10. Papuamides

**Figure 32:**
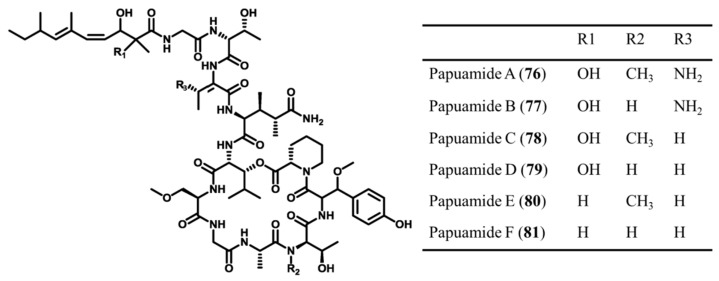
Structures of papuamide A–F (**76**–**81**) isolated [83,104,105].

Papuamide A–D (**76–79**), cyclic depsipeptides isolated from *Theonella mirabilis* and *T. swinhoei*, are known as the first marine-derived peptides to effectively inhibit the infection of human T-lymphoblastoid cells by HIV-1 in vitro (Figure 32) [83,104]. Especially, papuamides A (**76**) and B (**77**) blocked the infection with an EC_50_ value of about 4 ng/mL and papuamide A has an average IC_50_ of 75 ng/mL, indicating cytotoxicity against a panel of human cancer cell lines. Papuamide E (**80**) and F (**81**) were isolated from *Melophlus* and LD_50_ values for brine shrimp were 104 and 106 μg/mL, respectively, confirming cytotoxicity [105].

#### 3.3.11. Phakellistatins

**Figure 33:**
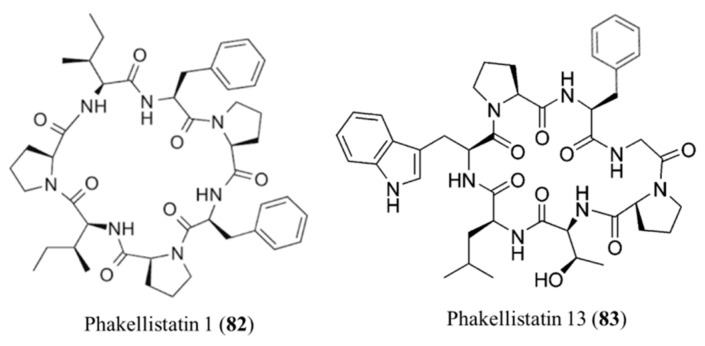
Structures of phakellistatin 1 (**82**) and 13 (**83**) [106].

Proline rich-cyclic heptapeptides, Phakellistatins, are isolated from *Phakellia carteri* and these compounds inhibit leukemia cell growth (Figure 33) [106]. Phakellistatin 1 (**82**) significantly inhibited growth of the P388 lymphocytic leukemia (50% effective dose, ED_50_ = 7.5 μg/mL) and other different melanoma cell lines [107]. Phakellistatin 13 (**83**) isolated from *Phakellia fusca* showed cytotoxicity against the BEL-7404 human hepatoma cell line (IC_50_ < 10 ng/mL). When synthetic phakellistatins were tested, they were inactive, unlike natural products. This may be explained by conformational differences, especially around the proline residue [108]. In other words, the biological properties of synthetic products were found to be significantly different from the products isolated from nature.

#### 3.3.12. Rolloamide A and B

**Figure 34:**
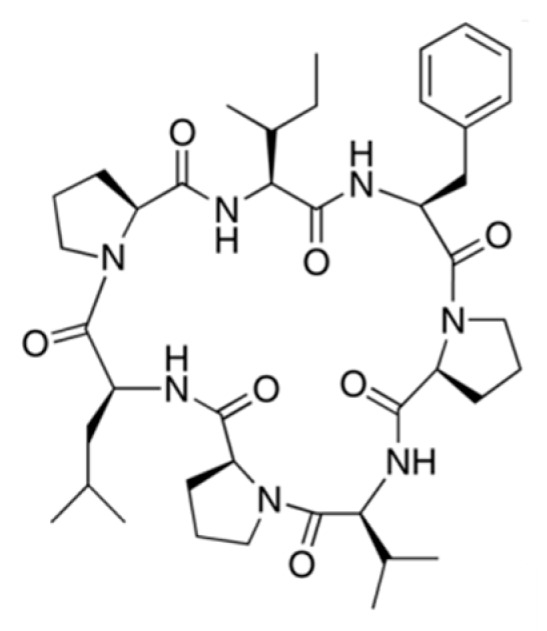
Rolloamide A (**84**) isolated from the Dominican sponge *Eurypon laughlini* [109].

Rolloamide A (**84**) and B are two cyclic heptapeptides contained in the Dominican sponge *Eurypon laughlini*. Rolloamide A (**84**) showed antiproliferative activity against a panel of histologically diverse cancer cells and showed moderate activity in prostate (LNCap and DU145), breast (MDA468, MDA435 and so on), ovarian (OVCAE3 and SKOV3) and diverse cell lines with IC_50_ value range from 0.4 to 5.8 μM, while rolloamide B was largely inactive (Figure 34) [109]. Rolloamide B has been shown to have potent activity against several gram-negative bacteria along with high antifungal potency [109].

#### 3.3.13. Scleritodermin A

**Figure 35:**
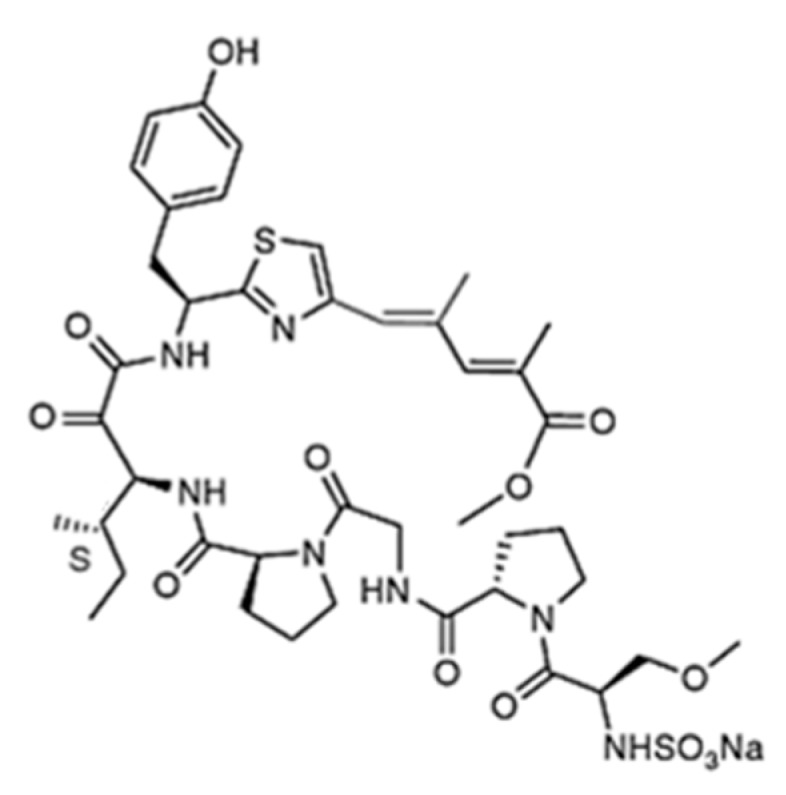
Structure of scleritodermin A (**85**) [110,111].

Scleritodermin A (**85**) isolated from *Scleritoderma nodosum* is a cyclic peptide and showed in vitro cytotoxicity against human tumor cell lines, including HCT116 colon carcinoma (IC_50_ = 1.92 μM), A2780 ovarian carcinoma (IC_50_ = 0.94 μM), and SKBR3 breast carcinoma (IC_50_ = 0.97 μM) (Figure 35) [110,111]. Scleritodermin A has a structure that combines the keto-Ile-Pro-Ser-Pro-SerOMe portion and the conjugated thiazole moiety. Scleritodermin A (**85**) has been observed to potently inhibit tubulin polymerization in different cancer cells because of the cell cycle arrest in the G2/M phase [110,111].

### 3.4. Tunicate and Ascidia-Derived Peptides

Marine tunicates and ascidiae produce many biologically active peptides; they produce more antitumor and anticancer peptides than any other group of marine organisms [7,11].

#### 3.4.1. Aplidin

**Figure 36:**
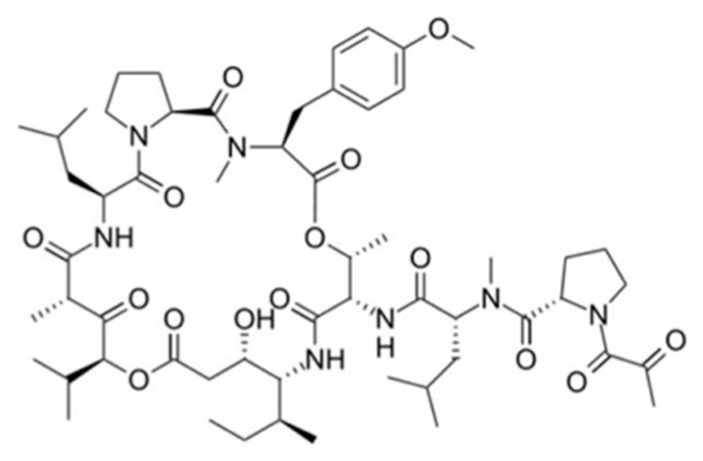
Structure of aplidin (**86**) [112].

Aplidin, the trade name for dehydrodidemnin B/plitidepsin (**86**), is a cyclic depsipeptide isolated from the ascidian *Aplidium albicans* (Figure 36) [112]. It has shown strong anticancer activity against a variety of human cancer cell lines including melanoma, breast and lung [113,114,115]. The toxicity of aplidine in normal hematopoietic tissue (IC_50_ = 150–2250 nM) was lower than that in tumor cells (IC_50_ = 0.2–27 nM).

The mechanism of action of aplidin involves cell cycle arrest at the G1-G2 phase, inhibition of protein synthesis, and induction of cancer cell death through induction of apoptosis [115]. Aplidin (**86**) induced early oxidative stress and then, the activation of both JNK and p38 mitogen-activated protein kinases (MAPK) occurred rapidly and steadily. Phosphorylation by the activation of both kinases occurred rapidly and fully in the drug treatment of HeLa human tumor cells. Activation of JNK and p38 MAPK resulted in downstream of cytochrome c release, upstream of caspase-9 and caspase-3 activation, and PARP cleavage, indicating the strong suppressor of the mitochondrial apoptosis pathway [115,116]. The two mechanisms (apoptosis via caspase-dependent and -independent mechanisms) by which aplidin activated JNK are rapid induction of Rac1 small GTPase and downregulation of MKP-1 phosphatase. Furthermore, aplidin induced other kinases such as the epidermal growth factor receptor (EGFR), the non-receptor protein-tyrosine kinase Src, and the serine/threonine kinases JNK and p38 MAPK in the MDA-MB-231 breast cancer cells.

The availability of the natural marine resource of aplidin is limited due to the difficulty of collecting *A. albicans*. The limited availability has led to the development of the synthetic analogues.

In patients with advanced melanoma, multiple myeloma, non-Hodgkin’s lymphoma, advanced medullary thyroid carcinoma, or urothelium carcinoma, relevant anticancer activity of plitidepsin has been shown in Phase III clinical studies [117,118,119,120].

Aplidin also inhibited the expression of the vascular endothelial growth factor gene, resulting in biological effects such as angiogenesis, hematopoiesis, and vascular homeostasis [121]. However, aplidin was more effective than didemnin B in preclinical models and, to date, there is no evidence of toxicity to life-threatening neuromuscular disease [112,115].

#### 3.4.2. Didemnin B

Some peptides derived from natural marine resource are shown to induce apoptosis through various mechanisms including cell membrane blebbing, nuclear condensation, and DNA fragmentation. However, the actual mechanism of action for this cytotoxic activity is still unclear. The cycle depsipeptides didemnins were first isolated from *Trididemnum solidum*, which showed strong antitumor, immunosuppressive, and antiviral properties [8,83].

**Figure 37:**
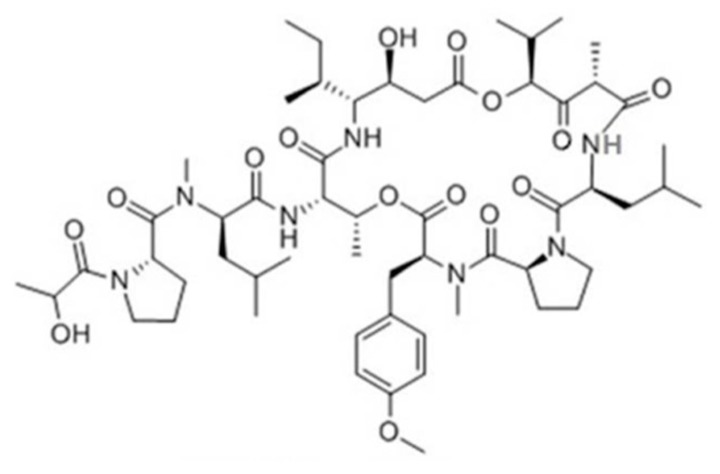
Structure of didemnin B (**87**) [123].

Didemnin B (**87**) showed the highest anticancer activity against human prostatic cancer cell lines (IC_50_ = 2 ng/mL in L1210 leukemia cell) and antiproliferative activity against P388 leukemia as well as B16 melanoma (Figure 37) [83]. For this reason, didemnin B (**87**) became the first ascidiacea cytotoxin to enter into clinical trials as a potent anticancer drug [122].

Didemnin B has shown impressive anti-cancer activity via the inhibition of protein synthesis [123]. Didemnin B has been approved for clinical use as an anticancer agent, but its use is limited due to toxicity and lack of efficacy during a phase II study and an unclear mechanism of action [124]. Furthermore, its low solubility and a short lifespan led to the termination of didemnin B phase II clinical trials [122]. Dehydrodidemnin B (aplidin), an oxidative derivatve of didemnin B, was isolated from *A. albicans* and exhibits more potent anticancer activity than didermin B [25]. Currently dehydrodidemnin B is in phase II clinical trials in the USA and Europe [25].

#### 3.4.3. Cycloxazoline

**Figure 38:**
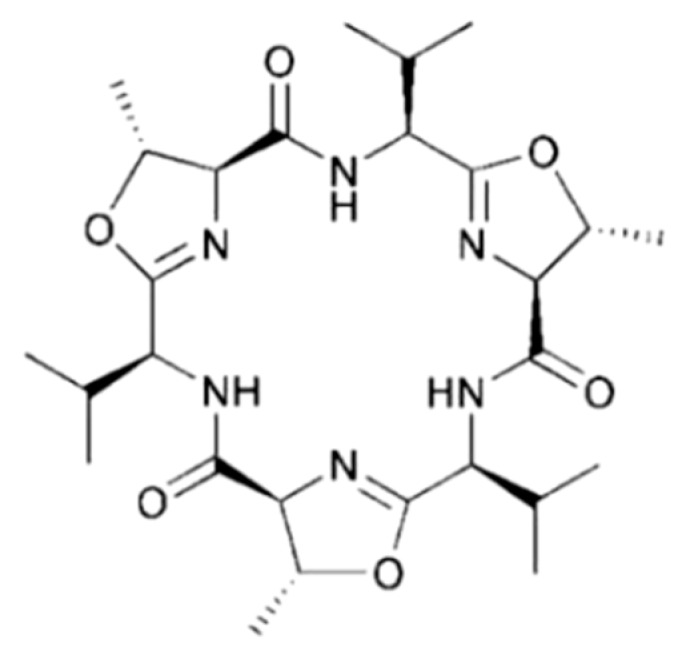
Cycloxazoline (**88**) isolated from ascidian *Lissoclinum bistratum* [124,125].

Cycloxazoline (**88**), a cyclic hexapeptide found from *Lissoclinum bistratum*, showed potent anti-apoptotic activity in various cancer cell lines but the exact target is presently unknown (Figure 38) [124,125]. The compound displayed cytotoxicity against MRC5CV1 and T24 cells (IC_50_ = 0.5 μg/mL). Cycloxazoline caused accumulation of HL-60 leukemia cells in the G2/M phase and interference with cytokinesis by phosphorylation of cellular proteins involved in cell-cycle control [125].

#### 3.4.4. Diazonamide A

**Figure 39:**
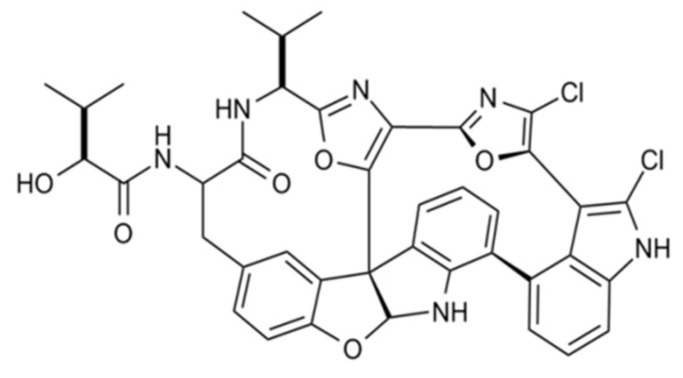
Diazonamide A (**89**) isolated from ascidian *Diazona angulata* [126,127].

Diazonamide A (**89**), a complex cytotoxic peptide was isolated from *Diazona angulate*, and displayed potent inhibition of tubulin polymerization in several cancer cells (Figure 39) [126,127]. It showed strong antitumor activity with an IC_50_ value of 2–5 nM against four human cancer cell lines (CA46, MCF7, PC-3, and A549). Furthermore, diazonamide A has a unique binding site for tubulin that differs from the vinca alkaloid or dolastatin 10 binding sites. So, it weakly inhibits the polymerization of tubulin into strong microtubules ends [126,127]. 

#### 3.4.5. Mollamide B and C

**Figure 40:**
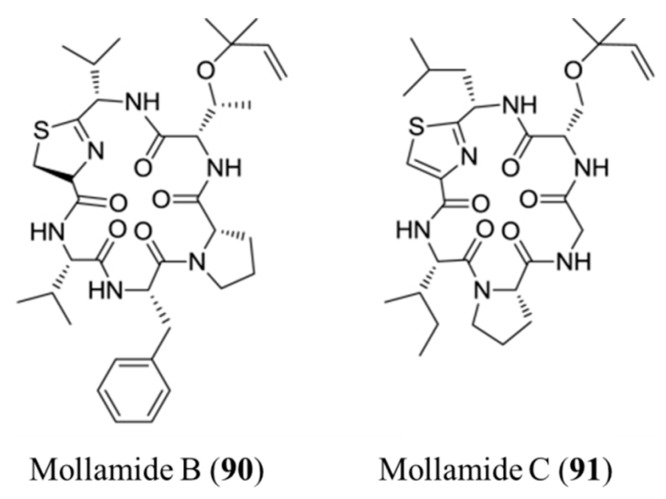
Mollamides B (**90**) and C (**91**) isolated from the ascidian *Didemnum molle* [128,129].

Mollamide from *Didemnum molle* was cyclodepsipeptide and showed cytotoxicity in P388 murine leukemia cells (IC_50_ values of 1 μg/mL) and in A549 human lung carcinoma and HT29 human colon carcinoma cells (2.5 μg/mL) (Figure 40) [128,129]. Among these, mollamide B (**90**) showed significant percent growth inhibition at 100 μM in the H460 non-small cell lung cancer cell, MCF7 breast cancer cell, and SF-268 CNS cancer cell line, but the National Cancer Institute (NCI) 60-cell line panel lacked tumor cell selectivity [130]. In contrast to mollamide B, mollamide C (**91**) showed strong in vitro cytotoxicity against leukemias (L1210 and CCRF-CEM), solid tumors (38, HCT-116, H125, MCF-7, and LNCaP), and a human normal cell (hematopoietic progenitor cell, CFU-GM); this was observed using a disk diffusion assay. The results showed that the leukemia and normal cells were slightly toxic but showed a larger difference in the solid tumor group [130].

#### 3.4.6. Tamandarin A and B

**Figure 41:**
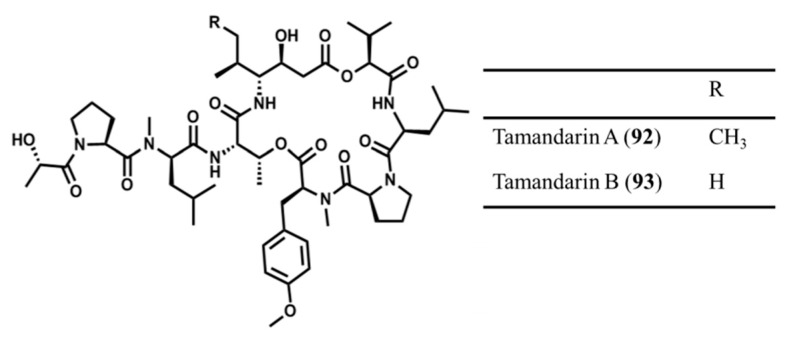
Chemical structure of tamandarin A (**92**) and tamandarin B (**93**) [131].

Tamandarin A (**92**) and B (**93**) are cyclodepsipeptides associated with didemnins known as highly active antiviral, antitumor, and immunosuppressive peptides [131]. Tamandarin A (**92**) and B (**93**) were isolated from *Trididemnum solidum*, *Trididemnum cyanophorum*, *Aplidium albicans*, and an unidentified ascidian (family Didemnidae) (Figure 41) [131]. Tamandarin A (**92**) and B (**93**) showed strong cytotoxicity against human cancer cell lines, and appeared to be a more potent inhibitor of protein synthesis in comparison with dedermin B [131]. However, their molecular mechanism of action is still unclear. Tamandarin A (**92**) was found to show cytotoxic activity on three cell lines, BX-PC3 pancreatic carcinoma, DU145 carcinoma, and UMSCC10b head and neck carcinoma with IC_50_ values of 1.79, 1.36, and 0.99 ng/mL, respectively [131]. 

#### 3.4.7. Trunkamide A

**Figure 42:**
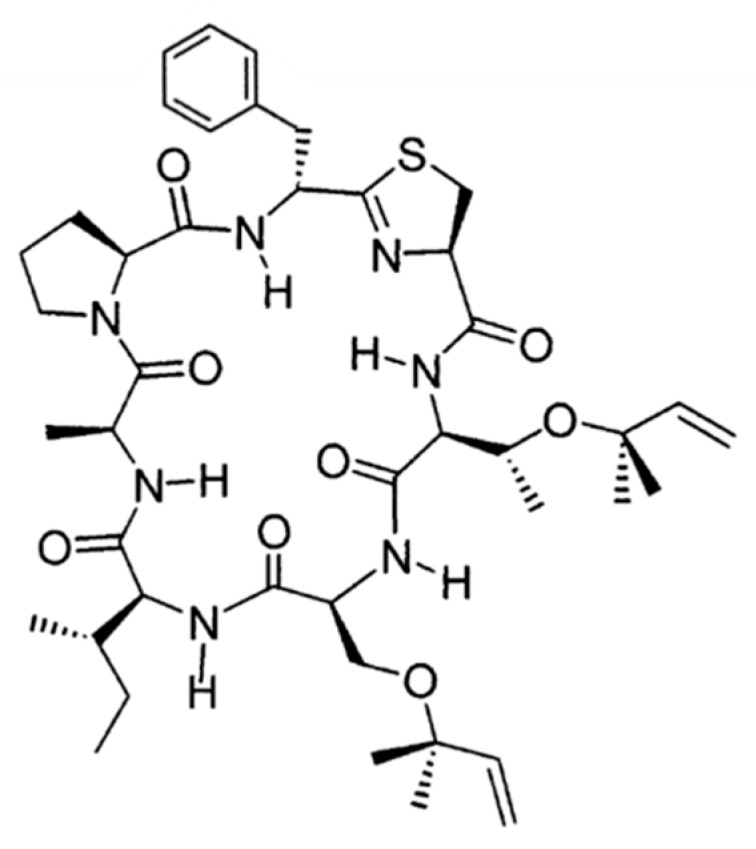
Trunkamide A (**94**) isolated from ascidians of the genus Lissoclinum [132].

Trunkamide A (**94**) is a cyclopeptide with a triazoline ring similar to mollamide, obtained from Lissoclinum (Figure 42) [132]. Trunkamide A exhibited cytotoxicity with an IC_50_ of 0.5 μg/mL against P388 mouse lymphoma, A-549 human lung carcinoma, and HT29 human colon cell lines, and 1.0 μg/mL for MEL-28 human melanoma cell lines.

#### 3.4.8. Virenamide A–C

**Figure 43:**
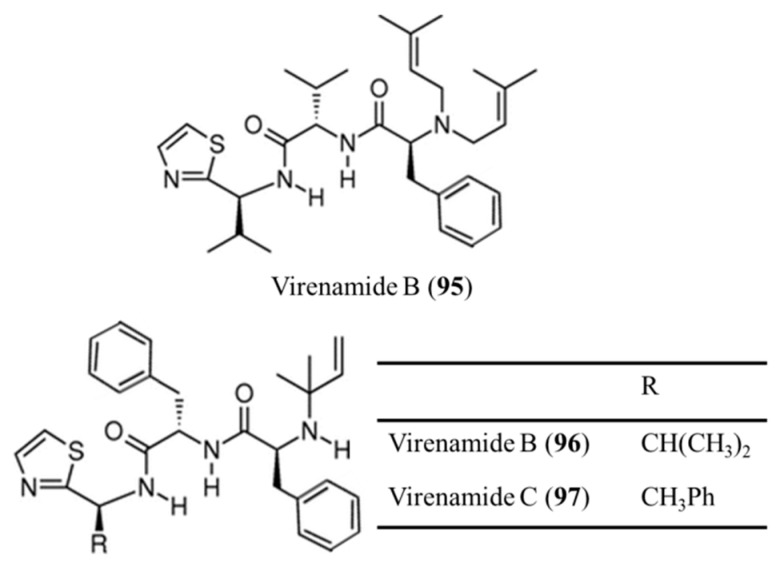
Structures of virenamide A–C (**95**–**97**) [133].

The linear cytotoxic tripeptides virenamide A–C (**95**–**97**) were isolated from *Diplosoma virens* and showed strong anti-apoptotic activity in several cancer cell lines (Figure 43) [133]. Virenamide A (**95**) had an IC_50_ of 2.5 μg/mL for P388 and 10 μg/mL for A549, HT29, and CV1 cells, and inhibited topoisomerase II activity. Both virenamide B (**96**) and C (**97**) had an IC_50_ of 5 μg/mL against P388, A549, HT29, and CV1 cells [133].

#### 3.4.9. Vitilevuamide

**Figure 44:**
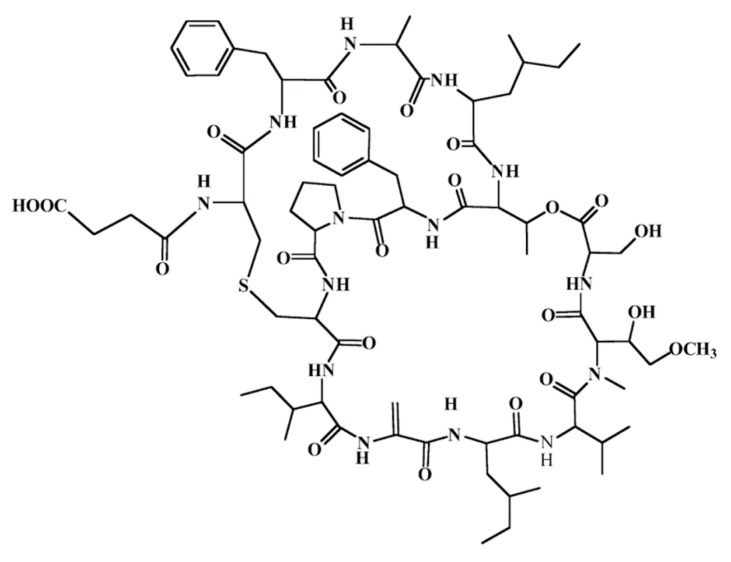
Structure of vitilevuamide (**98**) [134].

Vitilevuamide (**98**), a bicyclic depsipeptide isolated from *Didemnum cuculiferum* and *Polysyncranton lithostrotum*, is a tubulin-interactive agent, and potentially positive in a cell-based screening for tubulin polymerization inhibitors of purified tubulin (IC_50_ = 2.5 μM) (Figure 44) [134]. This demonstrates that vitilevuamide (**98**) showed non-competitive inhibition of vinblastine binding to tubulin.

The colchicine binding to tubulin was tested at concentrations up to 80 μM, and was found to be stabilized in the presence of vitilevuamide. It has also been shown to be weakly affected by the presence of GTP-binding in the presence of vitilevuamide, suggesting that vitilevuamide may inhibit tubulin polymerization through interaction at a unique site [134].

Vitilevuamide inhibited the growth of various human cancer cell lines with IC_50_ values in the range of 6–311 nM, confirming that they were associated with G_2_/M cell cycle arrest. In mice implanted with P388 lymphocytic leukemia, the intraperitoneal administration of vitilevuamide at doses of 12–30 mg/kg on days 1.5 and 9 after administration resulted in increased life span [122,134].

### 3.5. Mollusk and Fish-Derived Anticancer Peptides

Marine mollusks and fish-derived peptides are pharmacologically active products and often have activity in cancerous tumors. For this reason, many research groups have been interested in identifying anticancer peptides from fish for many years. However, there are few reports of marine mollusks and fish-derived anticancer peptides compared to other marine resources. Therefore, further research is needed to develop mollusks and fish-derived anticancer peptide and other physiologically active peptides, and it is expected to be a biomass in the future.

#### 3.5.1. Dolastatins

**Figure 45:**
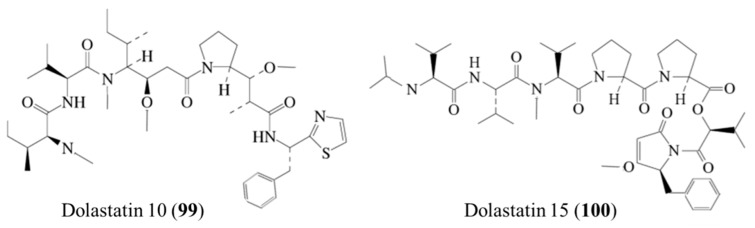
Chemical structure of dolastatin 10 (**99**) and dolastatin 15 (**100**) [137,138].

Dolastatins are cytotoxic linear or cyclic peptides isolated from *Dolabella auricularia*, among the dolastatins, dolastatin 10 (**99**) and 15 (**100**) showed the most promising antiproliferative action (Figure 45) [135]. Dolastatin 10 is a linear pentapeptide that contains several unique amino acid residues and is the most potent antiproliferative dolastatin. It inhibited the cell growth of L1210 murine leukemia (IC_50_ = 0.5 nM) and human bucket lymphoma CA46 cells (IC_50_ = 50 pM). For this reason, dolastatin 10 (**99**) has been selected for clinical trials because of its favorable preclinical advantage and is currently on clinical trials in Phase I as an anticancer drug [136].

The depsipeptide dolastatin 15 (**100**) inhibited the growth of L1210 cells (IC_50_ = 3 nM) and CA46 cells (IC_50_ = 5 nM) [137]. Dolastatin 10 was also extremely potent in vitro. Microtubule assembly and tubulin polymerization were inhibited, causing the accumulation of cells that were arrested in metaphase [138,139]. Dolastatin 10 is a promising candidate with an antineoplastic profile against various cancer cell lines. It has been investigated in various phase I clinical studies, which reported good tolerability and identified bone marrow depression as dose-limiting toxicity. However, dolastatin 10 has been found to cause myelosuppression, peripheral sensory neuropathy, and local irritation at the drug injection site. Structural complexity, low synthetic yield and low water solubility of dolastatins are critical barriers to their broadly clinical evaluation as anticancer drugs [137].

Based on the structural model of dolastatin 10, various analogs were synthesized. Among these analogs, TZT-1027 is a drug with antivascular activity that depolarizes microtubules and disrupts newly formed tumor vasculature. TZT-1027 was found to possess antiangiogenic activity in chorioallantoic membrane embryo (CAM) and human umbilical vein endothelial cells (HUVECs) in vivo [140]. However, it did not show anticancer activity in a phase II clinical trials in patients with advanced non-small cell lung cancer treated with platinum-based chemotherapy [141].

Dolastatin 15 (**100**) isolated from *D*. *auricularia* has not yet been clinically studied, but water-soluble derivates LU-103793 (cematodin) and ILX651 (synthadotin) were developed as cancer drug candidates for clinical studies. LU-103793 was successful in a phase I clinical trial for the treatment of several cancers. The phase II trial was interrupted by unexpected research results. ILX-651 successfully completed the phase I clinical trial and a phase II trial has been recommended owing to its good tolerability with no cardiotoxicities, unlike LU-103793. ILX651 has finished at the least three rounds of phase II clinical trials in patients with advanced and/or metastatic hormone-refractory prostate cancer [29].

#### 3.5.2. Kahalalides

**Figure 46:**
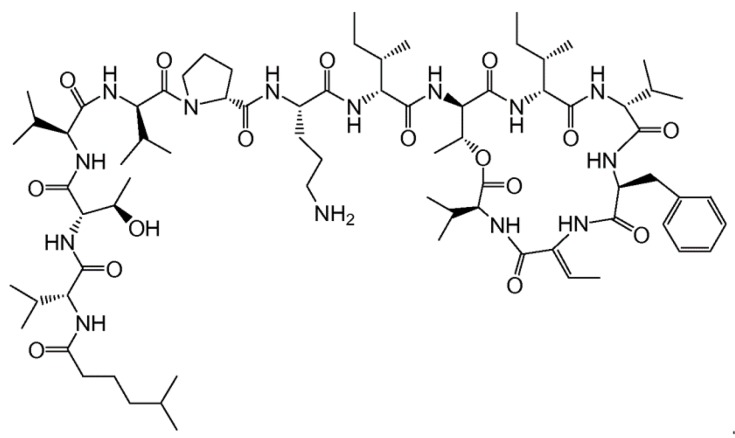
Structure of kahalalide F (**101**) [142].

Kahalalides are peptides isolated from *Elysia rufescens*. Among the kahalalides, kahalalide F (**101**) is a depsipeptide [142], displaying both in vitro and in vivo antitumor activity in colon (IC_50_ = 0.162–0.288 μM), A549 breast (IC_50_ = 0.135 μM), H5578T non-small cell lung (IC_50_ = 0.162 μM), and HS-578T-particular prostate cancer (IC_50_ = 0.479 μM) (Figure 46) [142,143,144].

Kahalalide F exhibits various mechanisms, such as lysosomal membrane, inhibition of ErbB (HER3) receptor tyrosine kinase, induction of oncosis, influence on the cell membrane permeability, and mediation of necrosis-like apoptosis [142,143,144,145,146,147,148]. Furthermore, kahalalide F protects tumor cell growth and spreading by inhibiting the expression of specific genes that trigger DNA replication and cell proliferation [149]. Extensive data indicated that kahalalide F exhibits strong cytotoxicity against lung non-small cell carcinoma (A549), melanoma, androgen-independent prostate cancer, hepatocellular carcinoma (HepG2), colon cancer (LoVo), and breast cancer (SKBR-3, BT474, MCF7, and MDA-MB-231) cell lines [149]. Kahalalide F is currently in phase I clinical trials for advanced malignant melanoma patients [150].

A synthetic cyclic depsipeptide kahalalide F derivative, elisidepsin (PM02734, also known as Irvalec^®^) has potential antineoplastic activity similar to kahalalide F. Elisidepsin shows anti-proliferative activity in a broad variety of cancer cell types [151,152,153]. The action of elisidepsin appears to be as a result of the induction of oncolysis rather than cell death by apoptosis in cancer cells. Elisidepsin is also in phase II clinical trials on metastatic or advanced gastroesophageal cancer [154]. These results were promising and presented a rational basis for further investigations and clinical trials for cancer treatment [155].

#### 3.5.3. Keenamide A

**Figure 47:**
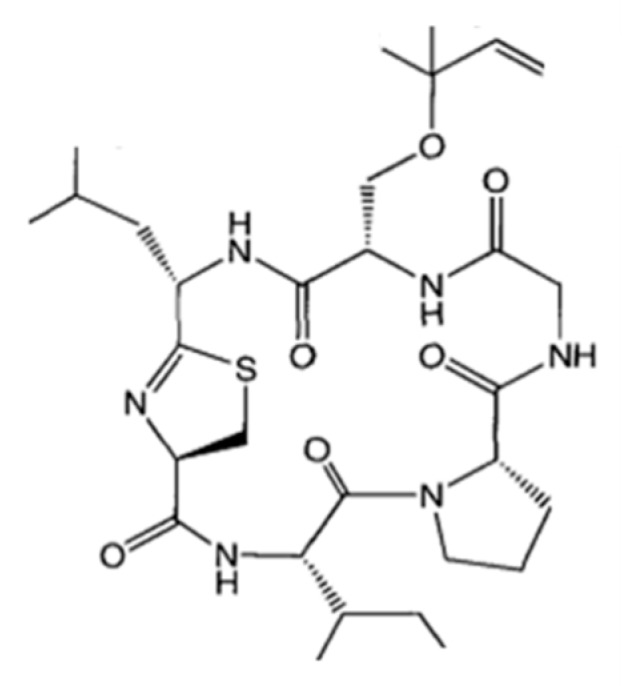
Structure of keenamide A (**102**) [156].

Keenamide A (**102**) is a cyclic hexapeptide isolated from *Pleurobranchus forskalii*, which has been shown to cause antitumor activity through undiscovered mechanisms (Figure 47) [156]. Keenamide A (**102**) showed anticancer activity against several tumor cell lines such as P-388, A-549, MEL-20 (IC_50_ = 2.5 μg/mL) and HT29 (5.0 μg/mL). In addition, the anti-malarial activity for D6 and W2 clones of *Plasmodium falciparum* was tested using keenamide A but showed no significant activity [156].

#### 3.5.4. Kulokekahilide-2

**Figure 48:**
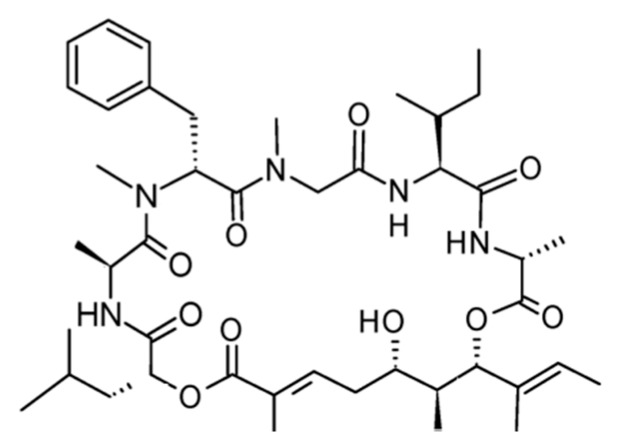
Structure of kulokekahilide-2 (**103**) [157].

The cyclic depsipeptide, kulokekahilide-2 (**103**), is the potent aurilide-related substance first isolated from *Philinopsis speciosa* and it is biosynthesized by marine cyanobacteria owing to its structural similarities with the aurilides (Figure 48) [157]. Kulokekahilide-2 (**103**) showed strong antitumor activity against various cell lines, such as P388, SKOV-3, MDA-MB-435, and A-10 (IC_50_ = 4.2~59.1 nM) [157].

#### 3.5.5. Ziconotide

**Figure 49:**
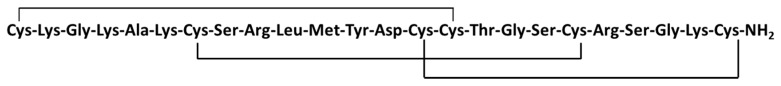
Primary structure of ziconotide (**104**) [158]. Ziconotide has six cysteine residues, forming three disulfide bonds.

Ziconotide (**104**) present in the venom of *Conus magus*, has a structure in which 25 amino acid peptides linked by three disulfide bonds (Figure 49) [158]. Ziconotide has been developed as an atypical analgesic to relieve severe and chronic pain by acting as an optional *N*-type voltage gated calcium channel blocker. This action has been shown to relieve pain by inhibiting the release of glutamate, calcitonin gene related peptide and pro-invasive neurochemicals such as substance P in the brain and spinal cord [158,159].

It has been shown to have a 1000-fold higher activity than morphine in animal models of nociceptive pain and has a remarkable analgesic character [158]. Ziconotide (trade name Prialt^®^) was the first marine peptide to receive FDA approval for analgesic use in 2004 and another marine peptide Brentuximab vedotin (trade name Adcetris^®^) was approved by the FDA in 2011 for drugs that are effective against cancer [159,160]. Various marine peptides are now entering phases of clinical trials in the United States and Europe [161].

#### 3.5.6. Pardaxin

Pardaxin (**105**, GFFALIPKIISSPLFKTLLSAVGSALSSSGGQE-NH_2_) is an antimicrobial peptide consisting of 33 amino acids, isolated from the fish *Pardachirus marmoratus* [77]. This peptide showed antitumor activity in human fibrosarcoma (HT1080) cells and HeLa cells, which inhibited proliferation in a dose-dependent manner in HT1080 cells and induced programmed cell death in HeLa cells [77]. In HT1080 cells, pardaxin induced apoptosis by causing caspase-dependent and ROS-mediated cell death [162].

Pardaxin plays important roles in the scavenging of reactive oxygen species (ROS) to alleviate c-Jun activation. Small interfering RNA-mediated knockdown of c-Jun requiring ROS and c-Jun in pardaxin-induced apoptosis signaling as it abrogates pardaxin-induced caspase activation and cell death [162].

Pardaxin, unlike HT1080 cells, causes caspase-dependent and caspase-independent apoptosis in human cervical cancer cells. Pardaxin also induces >90% inhibition of colony formation in MN-11 cells derived from MC1A fibrosarcoma in male C57BL/10 mice at a concentration of 13 μg/mL [163]. In addition, pardaxin has a potential veterinary application because it has a lytic action with potent activity against canine perianal gland adenomas [163].

#### 3.5.7. YALRAH

Tyr-Ala-Leu-Pro-Ala-His (**106**), an anticancer peptide consisting of six residues derived from the half-fin anchovy (*Setipinna taty*), has been shown to inhibit the proliferation of prostate cancer cells [164]. YALPAH and three analogs (YALRAH, YALPAR and YALPAG) exhibited anti-proliferative activity in PC3 cells. Among them, the modified peptide YALRAH showed the strongest activity (IC_50_ value of 11.1 μM). It has been confirmed that arginine (R) is an important residue for anticancer activity, but the mechanism for this is still unclear [164].

## 4. Anticancer Peptide-Based Drug Therapeutics Developed from Marine Organisms and Future Prospects

Many factors are involved in the discovery and development of anticancer drugs from marine natural products. However, there are various reasons that have ensured the use of marine peptides in the search for anticancer drugs. Marine-derived peptides are chemically diverse, have a wide range of therapeutic activities, and are highly specific to cells or tissues. These peptides can act specifically against cancer cells by either membranolytic mechanisms or mitochondrial disruption [165]. Generally, the negative net charge of the cancer membrane is an important factor for peptide selectivity and toxicity, especially relative to the typically zwitterionic properties of normal cell membranes [166]. Amphiphilicity levels and hydrophobic arc size allow the penetration of these peptides through cancerous cell membranes, leading to destabilization of membrane integrity [167,168]. Since they are composed of metabolically and allergenically tolerable amino acids, the risk of undesirable destructive side-reactions is reduced, and they are usually safe and non-toxic compounds. Furthermore, anticancer peptides are also used as vehicles in drug formulations for the improvement of biological properties, targeted drug delivery, or transport through target cell membranes [169]. Several potential anticancer peptides, such as stimuvax, primovax, melanotan, and cilengitide, are in clinical trials [170]. Combination therapy has emerged as an important strategy for fighting cancer in recent years because a single method may not be enough to completely cure the disease and prevent recurrence [159]. For the purpose of synergistic effects, combinations of anti-angiogenic compounds and traditional therapies are currently being investigated in clinical trials and will make anticancer peptide discovery more interesting in the next few years.

## Figures and Tables

**Table 1:** List of marine-derived anticancer peptides and their mode of action.

Name of Peptide	Natural Sources	Class/Types	Mode of Action/Investigative Status	Growth Inhibition Concentration (Cell Line)	Ref.
Apratoxin A–D (**1**–**4**)	Cyanobacteria: *Lyngbya majuscula*, *L. sordida*	Cyclic depsipeptide	Induction of G1 phase cell cycle arrest and apoptosis/in vitro only	IC_50_: 0.36 nM (LoVo), 0.52 nM (KB), 2.6 nM (H-460)	[29,30,31,32,33,34,35]
Aurilide (**5**), B (**6**), C (**7**)	Cyanobacteria: *Lyngbya majuscula*	Cyclic depsipeptide	Cytotoxicity /in vitro only	LC_50_: 40–130 nM (NCI-H460), 10–50 nM (neuro-2a), GI_50_ < 10 nM (NCI 60 cell line panel)	[36,37]
Bisebromoamide (**8**)	Cyanobacteria: *Lyngbya majuscula*	Linear peptide	Activation of ERK pathway/in vitro only	IC_50_: 0.04 μg/mL (HeLa S3), GI_50_ = 40 nM (JFCR39 cell line panel)	[38]
Coibamide A (**9**)	Cyanobacteria: *Leptolyngbya* sp.	Cyclic depsipeptide	Cancer cell proliferation inhibition/in vitro only	LC_50_ < 23 nM (NCI-H460, neuro-2a) GI_50_ < 7.6 nM (NCI 60 cell line panel)	[39]
Cryptophycin (**10**)	Cyanobacteria: *Nostoc* sp.	Depsipeptide	Apoptosis and microtubule inhibition /in vitro only (Cryptophycin-52: Phase II human clinical trial)	IC_50_ < 50 pM (MDR tumor cell lines),	[40,41,42,43,44]
Desmethoxymajusculamide C (**11**)	Cyanobacteria: *Lyngbya majuscula*	Cyclic depsipeptide	Tubulin polymerization Inhibition/in vitro only	IC_50_: 20 nM (HCT-116),	[45]
Grassypeptolide A–E (**12**–**16**)	Cyanobacteria: *Lyngbya confervoides*	Cyclic depsipeptide	Induction of G2/M phase cell cycle arrest /in vitro only	IC_50_: 192–335 nM (HeLa), 407–599 nM (neuro-2a)	[46,47]
Hantupeptin A (**17**)	Cyanobacteria: *Lyngbya majuscula*	Cyclic depsipeptide	Cytotoxicity /in vitro only	IC_50_: 32 nM (MOLT-4), 4 μM (MCF-7)	[48,49,50]
Hectochlorin (**18**)	Cyanobacteria: *Lyngbya majuscula*	Lipopeptide	Hyperpolymerization /in vitro only	IC_50_: 20 nM (CA46), 300 nM (PtK2)	[51]
Hormothamnin A (**19**)	Cyanobacteria: *Hormothamnion enteromorphoides*	Cyclic undecapeptide	Cytotoxicity /in vitro only	IC_50_: 0.13–0.72 μg/mL (SW1271, A529, B16-F10, HCT-116))	[52]
Itralamide A (**20**), B (**21**)	Cyanobacteria: *Lyngbya majuscula*	Cyclic depsipeptide	Antiproliferative activity/in vitro only	IC_50_: 6 μM (HEK293)	[53]
Lagunamide A–C (**22**–**24**)	Cyanobacteria: *Lyngbya majuscula*	Cyclic depsipeptide	Antiproliferative activities and apoptosis/in vitro only	IC_50_: 6.4–24.4 nM (P388)	[54,55]
Largazole (**25**)	Cyanobacteria: *Symploca* sp.	Cyclic depsipeptide	Stimulation of histone hyperacetylation in the tumor/in vitro only	GI_50_: 7.7 nM (MDA-MB-231), 122 nM (NMuMG), 55 nM (U2OS), 480 nM (NIH3T3)	[56,57,58,59,60]
Laxaphycin A (**26**), B (**27**)	Cyanobacteria: *Lyngbya majuscula*	Cyclic peptide	Antiproliferative activities/in vitro only	IC_50_ < 2 μM (CEM-WT)	[61,62,63]
Lyngbyabellin A (**28**), E (**29**), B (**30**)	Cyanobacteria: *Lyngbya majuscula*	Lipopeptides	Cytoskeletal actin disruption/in vitro only	IC_50_: 0.03–0.1 μg/mL (KB), IC_50_: 0.5–0.83 μg/mL (LoVo), LC_50_: 0.4–1.2 μg/mL (NCI-H460, neuro-2a)	[64,65]
Lyngbyastatin 4–7 (**31**–**34**)	Cyanobacteria: *Lyngbya* sp.	Depsipeptide	Porcine pancreatic elastase inhibition /in vitro only	IC_50_: 120–210 μM (elastase inhibition)	[66,67]
Symplocamide A (**35**)	Cyanobacteria: *Symploca* sp.	Cyclic depsipeptide	Proteasome inhibition/in vitro only	IC_50_: 40 nM (NCI-H460), 29 nM (neuro-2a)	[68]
Tasiamide (**36**), B (**37**)	Cyanobacteria: *Symploca* sp.	Linear peptide	Cytotoxicity /in vitro only	IC_50_: 0.48 μg/mL (KB), 3.47 μg/mL (LoVo)	[69,70,71]
Veraguamide A (**38**), D (**39**), E (**40**)	Cyanobacteria: *Oscillatoria margaritifera*, *Symploca* cf. *Hydnoides* sp.	Cyclic depsipeptide	Cytotoxicity /in vitro only	LC_50_: 141 nM (H-460), IC_50_: 0.5–1.5 μM (HT29, HeLa)	[72,73]
Azonazine (**41**)	Fungus: *Aspergillus insulicola*	Hexacyclic dipeptide	Cytotoxicity /in vitro only	IC_50_ < 15 ng/mL (HCT-116)	[74]
Sansalvamide A (**42**)	Fungus: the genus *Fusarium*	Cyclic depsipeptide	Apoptosis and inhibition of topoisomerase I /in vitro only	IC_50_: 4.5 μg/mL (HT29)	[75,76]
Scopularides A (**43**), B (**44**)	Fungus: *Scopulariopsis brevicaulis*	Cyclic depsipeptide	Cytotoxicity /in vitro only	IC_50_: 10 μg/mL (Colo357, Panc89, HT29)	[77]
Arenastatin A (**45**)	Sponge: *Dysidea arenaria*	Cyclic depsipeptide	Inhibition of microtubule assembly /in vitro only	IC_50_: 5 pg/mL (KB)	[78,79,80,81,82,83]
Discodermin A–H (**46**–**53**)	Sponge: *Discodermia kiiensis*	Tetra- decapeptide	Membrane permeabilization /in vitro only	IC_50_: 0.02–20 μg/mL (P388, A549)	[84]
Geodiamolide H (**54**)	Sponge: *Geodia corticostylifera*	Cyclic depsipeptide	Antiproliferative activity /in vitro only	G_100_: 18.6 nM (OV Car-4),	[85,86,87]
Hemiasterlin (**55**), A (**56**), C (**57**)	Sponge: *Auletta* sp., *Siphonochalina* sp.	Linear tripeptide	Inhibition of tubulin polymerization /Phase I human clinical trial, (HT1286: Phase I)	IC_50_: 0.0484–0.269 nM (PC3), 0.404–10.3 nM (NFF),	[88,89,90,91,92,93,94,95]
Homophymine A–E (**58**–**62**), A1–E1 (**63**–**67**)	Sponge: *Homophymia* sp.	Cyclic depsipeptide	Activation of caspase-3 and 7/in vitro only	IC_50_: 2–100 nM (MCF7/MCF7R, HCT116/HCT15, HL60/HL60R)	[96]
Jaspamide (**68**)	Sponge: *Jaspis johnstoni*	Cyclic depsipeptide	Activation of caspase-3, depression of Bcl-2 protein expression /in vitro only	IC_50_ : 0.04 ng/mL (P388)	[97,98,99]
Koshikamide B (**69**), F–H (**70**–**72**)	Sponge: *Theonella* sp.	Peptide lactone	Cytotoxicity /in vitro only	IC_50_: 0.45–2.3 μM (P388), 5.5–10 μM (HCT-116)	[100,101]
Microcionamide A (**73**), B (**74**)	Sponge: *Clathria* (*Thalysias*) *abietina*	Cyclic heptapeptide	Cytotoxicity /in vitro only	IC_50_: 125–177 nM (MCF-7), 98–172 μM (SKBR-3)	[102]
Orbiculamide A (**75**)	Sponge: *Theonella* sp.	Cyclic peptide	Cytotoxicity /in vitro only	IC_50_: 4.7 μg/mL (P388)	[103]
Papuamide A–F (**76**–**81**)	Sponge: *Theonella mirabilis*, *T. swinhoei*	Cyclic depsipeptide	Cytotoxicity and inhibition of infection /in vitro only	IC_50_: 0.75 ng/mL (human cell line panel)	[83,104,105]
Phakellistatin 1 (**82**), 13 (**83**)	Sponge*: Phakellia carteri*	Cyclic heptapeptides	Antiproliferative activity/in vitro only	ED_50_: 7.5 ug/mL (P388), IC_50_: 0.75 ng/mL (BEL-7404)	[106,107,108]
Rolloamide A (**84**)	Sponge: *Eurypon laughlini*	Cyclic heptapeptides	Tubulin polymerization inhibition/in vitro only	IC_50_: 0.4–5.8 μM (SKBR3, A2780)	[109]
Scleritodermin A (**85**)	Sponge: *Scleritoderm nodosum*	Cyclic peptide	Microtubule assembly inhibition/in vitro only	IC_50_ < 2 μM (HCT116, SKBR3, A2780)	[110,111]
Aplidin (plitidepsin, **86**)	Tunicate: *Aplidium albicans*	Cyclic depsipeptide	Activation of JNK and p38 MAPK/Phase III human clinical trial	IC_50_: 0.2–27 nM (CFU-GEMM, CFU-GM, BFU-E)	[112,113,114,115,116,117,118,119,120,121]
Didemnin B (**87**)	Tunicate: *Trididemnum solidum*	Cyclic depsipeptide	Apoptosis/Phase II human clinical trial	IC_50_: 2 ng/mL (L1210)	[122,123,124]
Cycloxazoline (**88**)	Ascidia: *Lissoclinum bistratum*	Cyclic hexapeptide	Apoptosis/in vitro only	IC_50_: 0.5 μg/mL (MRC5CV1, T24)	[125]
Diazonamide A (**89**)	Ascidia: *Diazona angulata*	Macrocyclic peptide	Tubulin polymerization inhibition/in vitro only	IC_50_: 2–5 nM (CA46, MCF7, PC3, A549)	[126,127]
Mollamide B (**90**), C (**91**)	Ascidia: *Didemnum molle*	Cyclic depsipeptide	Antiproliferative activity/in vitro only	IC_50_: 1 μg/mL (P388), 1 μg/mL (A549, HT29)	[128,129,130]
Tamandarin A (**92**), B (**93**)	Ascidia: *Didemnum* sp*.*	Cyclic depsipeptide	Cytotoxicity /in vitro only	IC_50_: 1.79 μg/mL (BX-PC3), 1.36 μg/mL (DU145), 0.99 μg/mL (UMSCC10b)	[131]
Trunkamide A (**94**)	Ascidia: *Lissoclinum* sp.	Cyclic peptide	Cytotoxicity /in vitro only	IC_50_: 0.5 μg/mL (P388, A549, HT29), 1.0 μg/mL (MEL-28)	[132]
Virenamides A–C (**95**–**97**)	Ascidia: *Diplosoma virens*	Linear tripeptides	Apoptosis /in vitro only	IC_50_: 5–10 μg/mL (P388, A549, HT29, CV1)	[133]
Vitilevuamide (**98**)	Ascidia: *Didemnum cuculiferum*, *Polysyncranton lithostrotum*	Bicyclic depsipeptide	Tubulin polymerization inhibition/in vitro only	IC_50_: 6–311 nM (P388, A549, HT29)	[122,134]
Dolastatin 10 (**99**), 15 (**100**)	Mollusk: *Dolabella auricularia*	Linear peptide	Microtubule assembly inhibition and Bcl-2 phosphorylation /Human clinical trial Dolastatin 10: phase II (TZT-1027: Phase III) Dolastatin 15: preclinical (ILX651: Phase II, LU-103793: Phase I)	IC_50_: 50–5000 pM (CA46), 0.5–3 nM (L1210)	[135,136,137,138,139,140,141]
Kahalalide F (**101**)	Mollusk: *Elysia rufescens*	Depsipeptide	ErbB3 protein and PI3K-Akt pathway involved in necrosis induction, apoptosis /Phase I human clinical trial (Elisidepsin: phase II)	IC_50_: 0.162–0.288 μM (colon), 0.135 μM (A549), 0.162 μM (H5578T), 0.479 μM (HS-578T)	[142,143,144,145,146,147,148,149,150,151,152,153,154,155]
Keenamide A (**102**)	Mollusk: *Pleurobranchus forskalii*	Cyclic hexapeptide	Cytotoxicity /in vitro only	IC_50_: 2.5 μg/mL (P388, A549, MEL-20), 5 μg/mL (HT29)	[156]
Kulokekahilide-2 (**103**)	Mollusk: *Philinopsis speciosa*	Cyclic depsipeptide	Cytotoxicity /in vitro only	IC_50_: 4.2–59.1 nM (P388, SKOV-3, MDA-MB-435, A-10)	[157]
Ziconotide (**104**)	Mollusk: *Conus magus*	Linear peptide	Selective *N*-type calcium channel blocker/FDA approved	IC_50_: 100 nM (HEK), 10 nM (IMR32)	[158,159,160,161]
Pardaxin (**105**)	Fish: *Pardachirus marmoratus*	Linear peptide	Caspase-dependent and ROS-mediated Apoptosis /active in animal	IC_90_: 13 μg/mL (NH-11)	[162,163]
YALRAH (**106**)	Fish: *Setipinna taty*	Linear peptide	Antiproliferative activity/in vitro only	IC_50_: 11.1 μM (PC3)	[164]
